# EasyClone‐MarkerFree: A vector toolkit for marker‐less integration of genes into *Saccharomyces cerevisiae* via CRISPR‐Cas9

**DOI:** 10.1002/biot.201600147

**Published:** 2016-06-23

**Authors:** Mathew M Jessop‐Fabre, Tadas Jakočiūnas, Vratislav Stovicek, Zongjie Dai, Michael K Jensen, Jay D Keasling, Irina Borodina

**Affiliations:** ^1^ The Novo Nordisk Foundation Center for BiosustainabilityTechnical University of DenmarkHørsholmDenmark; ^2^ The Novo Nordisk Foundation Center for BiosustainabilityChalmers University of TechnologyGothenburgSweden; ^3^ Department of Biology and Biological EngineeringChalmers University of TechnologyGothenburgSweden; ^4^ Joint BioEnergy InstituteEmeryvilleCAUSA; ^5^ Physical Biosciences DivisionLawrence Berkeley National LaboratoryBerkeleyCAUSA; ^6^ Department of Chemical and Biomolecular Engineering & Department of Bioengineering University of CaliforniaBerkeleyCAUSA

**Keywords:** CRISPR‐Cas9, 3‐hydroxypropionic acid, Metabolic engineering, *Saccharomyces cerevisiae*

## Abstract

*Saccharomyces cerevisiae* is an established industrial host for production of recombinant proteins, fuels and chemicals. To enable stable integration of multiple marker‐free overexpression cassettes in the genome of *S. cerevisiae*, we have developed a vector toolkit EasyClone‐MarkerFree. The integration of linearized expression cassettes into defined genomic loci is facilitated by CRISPR/Cas9. Cas9 is recruited to the chromosomal location by specific guide RNAs (gRNAs) expressed from a set of gRNA helper vectors. Using our genome engineering vector suite, single and triple insertions are obtained with 90–100% and 60–70% targeting efficiency, respectively. We demonstrate application of the vector toolkit by constructing a haploid laboratory strain (CEN.PK113‐7D) and a diploid industrial strain (Ethanol Red) for production of 3‐hydroxypropionic acid, where we tested three different acetyl‐CoA supply strategies, requiring overexpression of three to six genes each. Among the tested strategies was a bacterial cytosolic pyruvate dehydrogenase complex, which was integrated into the genome in a single transformation. The publicly available EasyClone‐MarkerFree vector suite allows for facile and highly standardized genome engineering, and should be of particular interest to researchers working on yeast chassis with limited markers available.

**Abbreviations**
Cas9: CRISPR associated protein 9
CRISPR: clustered regularly interspaced short palindromic repeats
DSB: double strand break (of DNA)
gRNA: guide RNA
3HP: 3‐hydroxypropionic acid
HR: homologous recombination
PAM: protospacer adjacent motif
USER: uracil‐specific excision reaction

## 1 Introduction

The yeast *S. cerevisiae* is widely applied in industrial biotechnology for production of fuels, chemicals, and pharmaceutical ingredients and it is also used for fundamental research as a eukaryotic model organism 1, 2, 3. Genetic manipulation of *S. cerevisiae* is greatly facilitated by its efficient inherent homologous recombination (HR) machinery for DNA double‐strand break (DSB) repair 4. As a result, many genome engineering tools have been developed that take advantage of HR for targeted integration of heterologous DNA into the yeast genome 5, 6. Chromosomal gene integration has several benefits over plasmid‐based approaches, such as increased strain stability, better control of gene expression level and lower population heterogeneity 5. However, though the HR machinery has a high fidelity, selection is still necessary to identify the colonies that have been successfully engineered. This has the fundamental drawback that selection markers need to be recycled if multiple genome edits are needed. Also, both dominant and auxotrophic markers have been reported to have an effect on cell physiology 7, 8. Moreover antibiotic‐based selection markers are undesirable in industrial strains due to the risk of spreading drug resistance. A way to improve homology‐directed chromosomal integration of heterologous gene fragments is by using DNA endonucleases for targeted DSB as this has been shown to increase recombination events by approximately three orders of magnitude 9, 10, 11, 12. Recently, the Clustered Regularly Interspaced Short Palindromic Repeats (CRISPR) Type II system from bacteria has been adopted for genetic engineering of yeast 13. This system consists of two components: guide RNA (gRNA) and CRISPR‐associated endonuclease (Cas9). Upon expression in the cell, the gRNA/Cas9 complex is recruited to DNA by base‐pairing of the gRNA recognition sequence (typically 20 bp) and the complementary DNA stand, after which the Cas9 introduces a DSB break. The target DNA sequence must be immediately followed by a protospacer adjacent motif (PAM) (i.e. NGG for *Streptococcus pyogenes*‐derived Cas9); Cas9 makes a blunt cut three nucleotides upstream of the PAM site 14. There have been many recently published examples of CRISPR‐Cas9‐based tools for yeast genome engineering 15, 16, 17, 18, 19, 20, 21 that are reviewed in Jakočiūnas et al. 22. The work presented here builds on the previously reported method EasyClone for single‐step targeted integration of multiple expression cassettes 5, 6. The EasyClone method allows targeted genomic integration of up to three vectors in a single transformation event using auxotrophic or dominant selection markers. These target sites are located in the intergenic regions and are interspaced by essential genes to ensure that the integrated DNA fragments are not at risk of being removed by HR 23. Moreover it has been confirmed that integration into these sites ensures a high‐level of expression and does not interfere with cellular growth 23. The selection markers can be removed via Cre‐LoxP‐mediated recombination, which requires one extra transformation with CreA‐vector, cultivation to induce the CreA expression and finally screening of the resulting clones on selection plates to confirm the loss of the marker; the marker removal procedure has a turnaround time of about five days. Once the selection markers are eliminated, the strain is ready for the next integration event or for other genetic modifications. Marker removal, as well as being time consuming, can also cause genome instability 24, 25.

Here we present the EasyClone‐MarkerFree method, which takes advantage of CRISPR‐Cas9 for introduction of double‐strand DNA breaks in the defined integration sites, which causes very efficient integration of expression cassettes and thus bypasses the need for selection. The gRNA helper vectors, expressing gRNA molecules that recruit Cas9 to the particular chromosomal locations, can be easily removed from the strain by growth on non‐selective medium, after which the strain is ready for the next modification. This shortens the turnaround time to only one or two days. Additional advantages are: simplified experimental design for cloning, because the choice of selection markers does not need to be taken into consideration; elimination of the effect of the selection markers on strain physiology; and moreover a possibility to iterate gene integrations with other edits guided by CRISPR/Cas9, e.g. gene deletions. The use of predefined and characterized chromosomal target sites dispenses with the need for the experimenter to identify suitable regions in which to integrate their genes of interest. Furthermore, we demonstrate the utility of the method by engineering 3‐hydroxypropionic acid producing strains with improved precursor supply strategies.

## 2 Materials and methods

### 2.1 Strains

Two different strains of *S. cerevisiae* were used: the haploid laboratory strain CEN.PK113‐7D (*MAT*a* URA3 HIS3 LEU2 TRP1 MAL2‐8* ^*c*^ *SUC2*; obtained from Peter Kötter, Johann Wolfgang Goethe University Frankfurt, Germany), and the diploid industrial Ethanol Red strain (*MAT*a/α; obtained from Fermentis, A Lesaffre division). *E. coli* strain DH5α alpha was used to clone, propagate, and store the plasmids.

### 2.2 Construction of plasmids

The codon‐optimized genes, encoding the pyruvate dehydrogenase complex and lipoate‐protein ligase from *Enterococcus faecalis*, were ordered from GenScript (sequences can be found in Supporting information, Table S5). The genes encoding ATP‐dependent citrate lyase and the mitochondrial citrate transport protein, were amplified from the genomic DNA of *Yarrowia lipolytica* DSM‐8218, obtained from the DSMZ collection (www.dsmz.de). All of the primers, biobricks and plasmids constructed and used in this study can be found in Supporting information, Tables S1–S3.

The EasyClone‐MarkerFree vectors were created by amplifying the EasyClone 2.0 vectors 6 with primers that were designed to attach to either side of the selection markers, creating a fragment that no longer contained the marker. These fragments were then ligated to form the marker‐less vectors. Seven of the resulting vectors (named ”Intermediate vectors“ in Table S3) contained PAM sites in the integration regions, which were removed by site‐directed mutagenesis using the QuikChange II XL Site‐Directed Mutagenesis Kit (Agilent Technologies) according to the manufacturers' protocol.

gRNA cassettes targeting particular integration loci (chromosomal coordinates can be found in Supporting information, Table S4) were ordered as double‐stranded gene blocks from IDT DNA. These cassettes were amplified using primers 10525(TJOS‐62 [P1F]) and 10529(TJOS‐65 [P1R]) and USER‐cloned into pCfB2926 (pTAJAK‐71) 15 to give single gRNA helper vectors. For construction of triple gRNA helper vectors, three gRNA cassettes were amplified using three primer pairs (10525(TJOS‐62 [P1F]) and 10530(TJOS‐66 [P2R]) for the first, 10526(TJOS‐63 [P2F]) and 10531(TJOS‐67 [P3R]) for the second, and 10527(TJOS‐64 [P3F]) and 10529(TJOS‐65 [P1R]) for the third gRNA cassette) and cloned into pCfB2926 (p‐TAJAK‐71) 15. Single gRNA helper vectors for Ethanol Red were constructed by PCR amplification of the template plasmid pCfB3041 using primers indicated in Supporting information, Table S1 as described in 17. All of the cloning steps for creating gRNA helper vectors and EasyClone‐MarkerFree vectors were performed in *E. coli*. Correct cloning was confirmed by Sanger sequencing.

For expression of the Cas9 gene we used an episomal vector pCfB2312 with CEN‐ARS replicon and KanMX resistance marker 17.

The EasyClone‐MarkerFree vectors for expression of fluorescent protein or 3HP pathway genes were cloned as described in 5, 26. The vectors were linearized with *Not*I, the integration fragment (part of the expression vector without *E. coli* ori and Amp^R^) was gel‐purified and transformed, along with a gRNA helper vector, into yeast carrying the Cas9 plasmid (pCfB2312) via the lithium acetate method 27. After the heat shock the cells were recovered for two hours in YPD medium and then plated on YPD agar containing 200 mg/L G418 and 100 mg/L nourseothricin. For yeast transformations with a single vector we routinely use 500 ng of the linear integration fragment along with 500 ng of the relevant gRNA helper plasmid. For yeast transformations with three vectors we use 1 µg of linear integration fragments and 1 µg of triple gRNA helper plasmid. Correct integration of the vectors into the genome was verified by colony PCR using primers listed in Supporting information, Table S1.

### 2.3 Media and growth conditions

The *E. coli* strains containing the plasmids were grown in Luria–Bertani (LB) media supplemented with 100 µg/mL ampicillin. Yeast strains were grown in liquid YPD media, or on YPD agar plates with 20 g/L glucose (Sigma‐Aldrich). When needed the medium was supplemented with antibiotics G418 (200 mg/L) for the selection of the Cas9 plasmid, and/or nourseothricin (100 mg/L) for selection of the gRNA plasmid. Synthetic complete medium with 20 g/L glucose was prepared using drop‐out powder from Sigma‐Aldrich.

The strains were screened for production of 3HP as following. Single colonies were grown in 500 µL synthetic complete media in 96 deep‐well plates with air‐penetrable lids (EnzyScreen, Germany) at 30°C with 250 rpm shaking overnight. The overnight cultures were then inoculated into 50 mL of either mineral media (MM) with 60 g/L glucose 5, or a simulated fed‐batch media (feed‐in‐time, FIT) in 500 mL‐shake flasks with baffles to a starting OD_600 _of 0.1. The feed‐in‐time (FIT) medium M‐Sc.syn‐1000 synthetic FIT was purchased from M2P labs GmbH (Germany). All the media for 3HP cultivations were supplemented with 500 ng/mL of DL‐α‐lipoic acid (Sigma Aldrich). The strains were cultivated in MM or FIT media at 30°C with 250 rpm for 168 h. Samples were taken every 12 h (every 1–2 h during the exponential phase in MM), with the optical density OD_600_ being measured on a 20‐fold diluted culture broth. Samples were then centrifuged at 3000 ×*g* for 30 min and the supernatant was stored at −20°C until HPLC analysis. Cultivations were carried out in triplicate.

### 2.4 Analytical procedures

Strains expressing *gfp* were analyzed using a BioTek Synergy MX microplate reader. Excitation wavelength was set at 485 nm, and emission was read at 530 nm. Optical density was measured at 600 nm. Concentration of 3‐hydroxypropionic acid in the broth was measured by HPLC, using a method previously reported in 28.

### 2.5 Integration Stability

Strains that had been transformed with *gfp*‐containing vectors were grown in 500 µL of YPD in 96 deep‐well plates with air‐penetrable lids at 30°C with 250 rpm shaking overnight. The following morning, 50 µL of each culture was passed to 500 µL of fresh YPD. This was repeated for a total of five passages. At the end of the passages, the culture was plated onto YPD agar, and the fluorescent colonies were counted.

### 2.6 Determination of gene copy number by qPCR

Quantitative PCR was performed on the Ethanol Red strain containing the genes from *Enterococcus faecalis*. Primers were designed using the PrimerQuest^®^ tool (https://eu.idtdna.com/Primerquest/Home/Index). Details of the primers used can be found in Supporting information, Table S1. Genomic DNA was prepared using the ZR Fungal/Bacterial DNA MiniPrep™ kit (Zymo Research) according to the manufacturer's protocol. Each reaction contained 10 µL of 2xSYBR Green qPCR MasterMix (Life Technologies), 1 µL of each primer, 0.1 µL of diluted reference dye, and 7.7 µL of genomic DNA. Reactions were carried out in triplicate, with 5 pg of gDNA present in each reaction. *ALG9* was used as a control, as well as a no template control where the gDNA was replaced with nuclease‐free PCR grade water. The experiment was carried out using a Stratagene Mx3005P qPCR system with the following settings; 10 min pre‐incubation at 95°C, 40 cycles of denaturation at 95°C for 20 s and annealing and elongation at 60°C for 22 s, and the final step of 95°C for 1 min, 55°C for 30 s, 95°C for 30 s. The Stratagene MxPro software was used to quantify the original amount of DNA present for each gene, which could then be related to the copy number.

## 3 Results and discussion

To enable rapid iterative cycles of metabolic engineering, we developed a new vector toolkit EasyClone‐MarkerFree, which allows integration of genes into specific sites of the yeast chromosome without integration of a selection marker. This simplifies strain design and reduces the turnaround time in comparison to the previously published EasyClone methods 5, 6. The method employs CRISPR‐Cas9 to introduce one or several DSBs into the host chromosome, ensuring efficient integration. For this system to be active in *S. cerevisiae*, it is necessary to have a Cas9 expression plasmid, as well as a gRNA helper vector. The DSB is repaired by the host cell using the native HR machinery, which incorporates the EasyClone‐MarkerFree vector(s) into the target loci via double cross‐over.

### 3.1 Construction of EasyClone‐MarkerFree vector toolkit

Construction of the EasyClone vector set required that 11 gRNA helper vectors were built, one to target each of the EasyClone chromosomal integration sites in the *S. cerevisiae* CEN.PK strain. Furthermore, three triple gRNA helper vectors were built that can target three sites simultaneously. The EasyClone 2.0 vectors were modified to remove the selection markers and, where necessary, to mutate the PAM sites in the integration sites. The vectors contain a USER cloning site surrounded by bi‐directional terminators. Genes and promoters can be cloned into the vectors using USER cloning as described below or other methods, such as Gibson assembly, in‐fusion, MoClo, etc. 29.

### 3.2 Workflow

**Figure 1:**
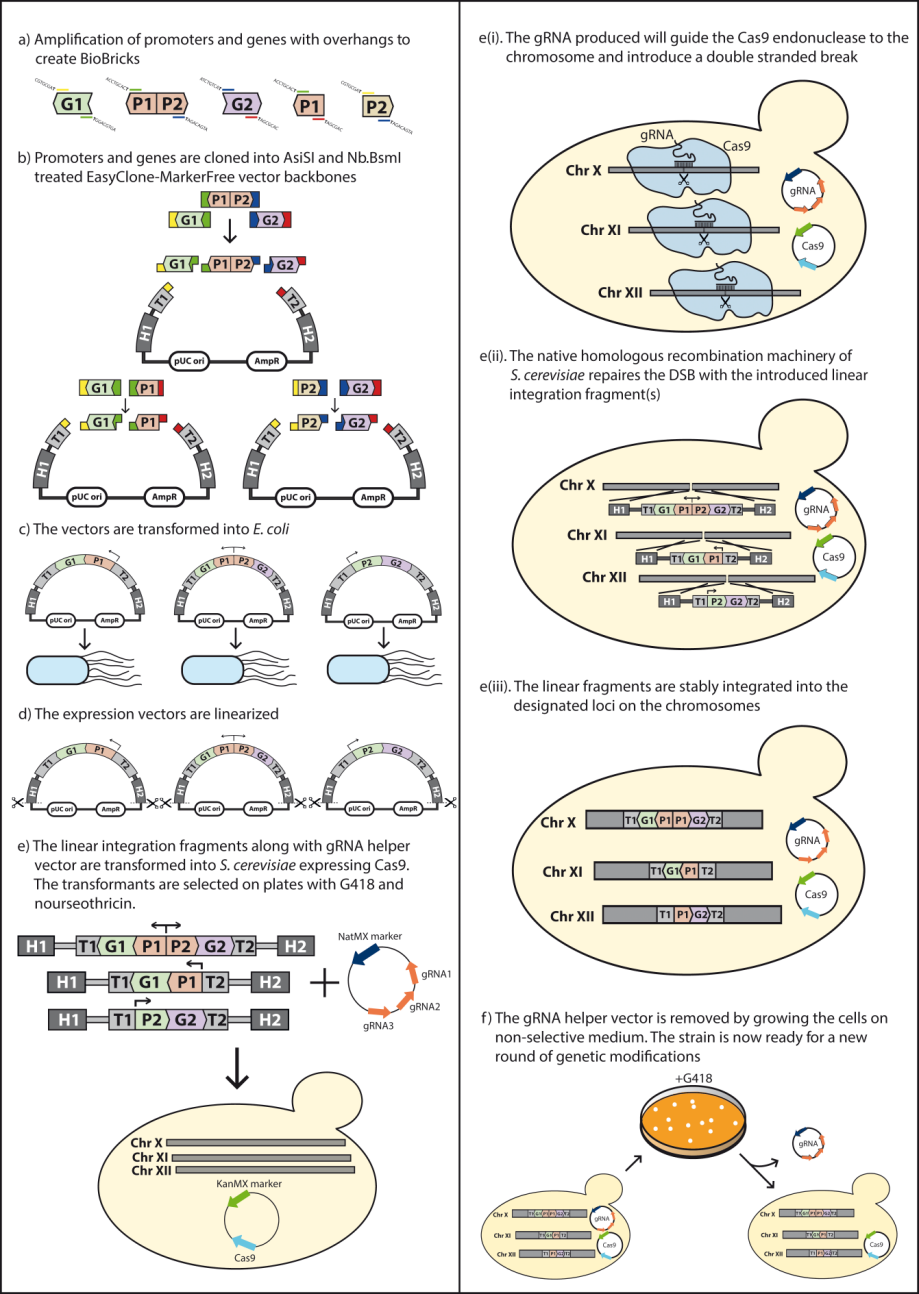
An overview of the EasyClone‐MarkerFree method. The BioBricks encoding genes and promoters are generated by PCR‐amplification with uracil‐containing primers (**a**). The BioBricks are assembled with the integration vectors via USER cloning (**b**) and the reaction mixture is transformed into *E. coli* (**c**). The resulting plasmids are isolated from *E. coli* and confirmed by sequencing, then they are linearized and transformed into Cas9‐expressing *S. cerevisiae* along with a helper gRNA vector, which causes double‐stranded breaks at the designated integration sites (**d–e**). Yeast cells are selected on plates with G418 and nourseothricin and correct integration of the vector(s) is confirmed by PCR. The helper gRNA vector can be eliminated by growth in the absence of nourseothricin selection and the resulting strain, which still expresses Cas9, can be further engineered. Once the desired genetic modifications are accomplished the Cas9 vector is removed by growth on non‐selective medium and the final strain, not containing selection markers, is obtained (**f**). G, gene; P, promoter; T, terminator; H, homology region. A detailed protocol is provided in Supporting information.

The suggested workflow is illustrated on Fig. 1. For USER cloning, the EasyClone‐MarkerFree vectors are prepared by linearization with *Asi*SI and nicking with Nb.*Bsm*I in order to obtain vector backbones with sticky ends. The treated vector backbones are stored at −20°C. Compatible biobricks, encoding genes and promoters, are prepared by PCR amplification using oligos with a uracil base, 7–10 bases downstream of the 5' end of the primers (Fig. 1a). We routinely use the overhangs as specified in 5 for all of our genes and promoters, which simplifies standardization and exchange of biobricks between scientists in our laboratory. We store the library of ready‐to‐use genes and promoters at −20°C.

To clone promoters and genes into the expression vector, the *Asi*SI/Nb.*Bsm*I‐treated vector backbones are mixed with the desired promoter(s) and gene(s) and treated with USER enzyme. The USER enzyme excises uracils in the overhangs of genes and promoters, thus producing sticky ends compatible with the EasyClone vectors (Fig. 1b). The USER reaction mix is transformed into *E. coli*, which ligates the expression vector (Fig. 1c). The plasmids can then be isolated, confirmed by sequencing and linearized with *Not*I (or another suitable) restriction enzyme to produce linear integration fragments containing expression cassettes flanked with ~500‐bp‐long integration regions (Fig. 1d). The linear integration fragment(s) and the corresponding helper vector are then transformed into the yeast host cell already carrying a Cas9 expression plasmid (Fig. 1e). The transformants are selected on medium with G418 and nourseothricin to select for the Cas9 and gRNA helper vector, respectively. If further CRISPR‐Cas9 mediated engineering rounds are desired, the gRNA helper vector is removed by cultivating the cells in liquid or solid medium containing G418 to maintain the selection for Cas9 (Fig. 1f). gRNA helper vectors are 2µ‐based and according to our experience are easily lost by growing the cells overnight in liquid medium with G418 only. The resulting culture is diluted and plated on G418 plates to obtain single colonies. The loss of the gRNA helper vector is confirmed by replica plating onto nourseothricin agar plates. The strains are then ready for the next round of Cas9‐mediated gene insertions or other manipulations (e.g. knock‐outs, etc.). If no further genetic modifications are desired, the strains can be cultivated on non‐selective medium to eliminate both Cas9 and gRNA helper vectors.

**Figure 2:**
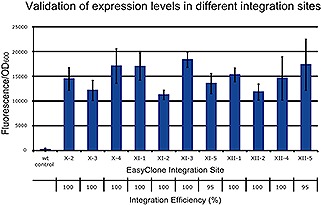
Validation of the EasyClone‐MarkerFree vector toolkit. The laboratory strain, CEN.PK 117‐7D, was transformed with the EasyClone‐MarkerFree vectors carrying *gfp* gene under control of a constitutive *P*_*TEF1*_ promoter. Specific fluorescence was measured to evaluate the expression level at each of the eleven integration sites. The error bars show standard deviation between nine or ten replicates. The percentage of clones with correct integration is shown for each site *n* = 20.

### 3.3 Validation of the vector toolkit

To verify the integration efficiency and gene expression levels at each site, *gfp* was cloned into each of the EasyClone vectors and transformed into the CEN.PK113‐7D strain. The integrations were tested by colony PCR for 20 randomly picked clones, and 95–100% correct integrations were obtained for all the integration sites (Fig. 2). This is comparable with the previous versions of EasyClone, where the targeting efficiency was 80–100% 5, 6. However, we should note that wrong clones in the EasyClone‐MarkerFree system were not fluorescent and hence they did not contain the designed integration cassettes in their genomes, while for the EasyClone system with selection markers, the integration cassette would always be integrated in the genome, however sometimes it would not be targeted to the designed integration site. Further, the transformed colonies were each checked for fluorescence, with 99% of the colonies showing fluorescence (84–559 colonies were obtained and tested per transformation). Fluorescence levels showed some smaller variations for different integration sites (Fig. 2) as we also observed previously 6. Another observation was that among those clones with an integrated fragment, all clones were fluorescent, which differs from the results we obtained with CRISPR‐Cas9‐mediated insertion of expression fragments with short homology arms (40 bp), where only half of the clones were fluorescent 17. We suggest that the high fidelity of correct integration is due to the long homology arms of ~0.5 kb present in the EasyClone vectors.

We further tested the stability of the integrations of the *gfp* gene in each of the sites. After five passages, the population retained 100% of fluorescent colonies, reinforcing the notion that these sites are stable due to their location in‐between essential genes.

The simultaneous targeting of three integration sites, assisted with triple gRNA helper vectors, resulted in 60–70% targeting efficiencies, which is also comparable with previously reported efficiencies for CRISPR‐Cas9 methods for multi‐loci integration 16, 18. We previously observed for the EasyClone system with selection markers that targeting efficiency into the same chromosome was only 44%, however the efficiency is higher for simultaneous integration into different chromosomes (unpublished results). We believe that simultaneous targeting of three sites that are closely positioned on the same chromosome, may lead to genome instability. Therefore to achieve the best targeting efficiencies, triple gRNA helper vectors were designed to target distinct chromosomes.

### 3.4 Application of EasyClone‐MarkerFree vector toolkit for engineering of precursor supply for 3‐hydroxypropionic acid production

**Figure 3:**
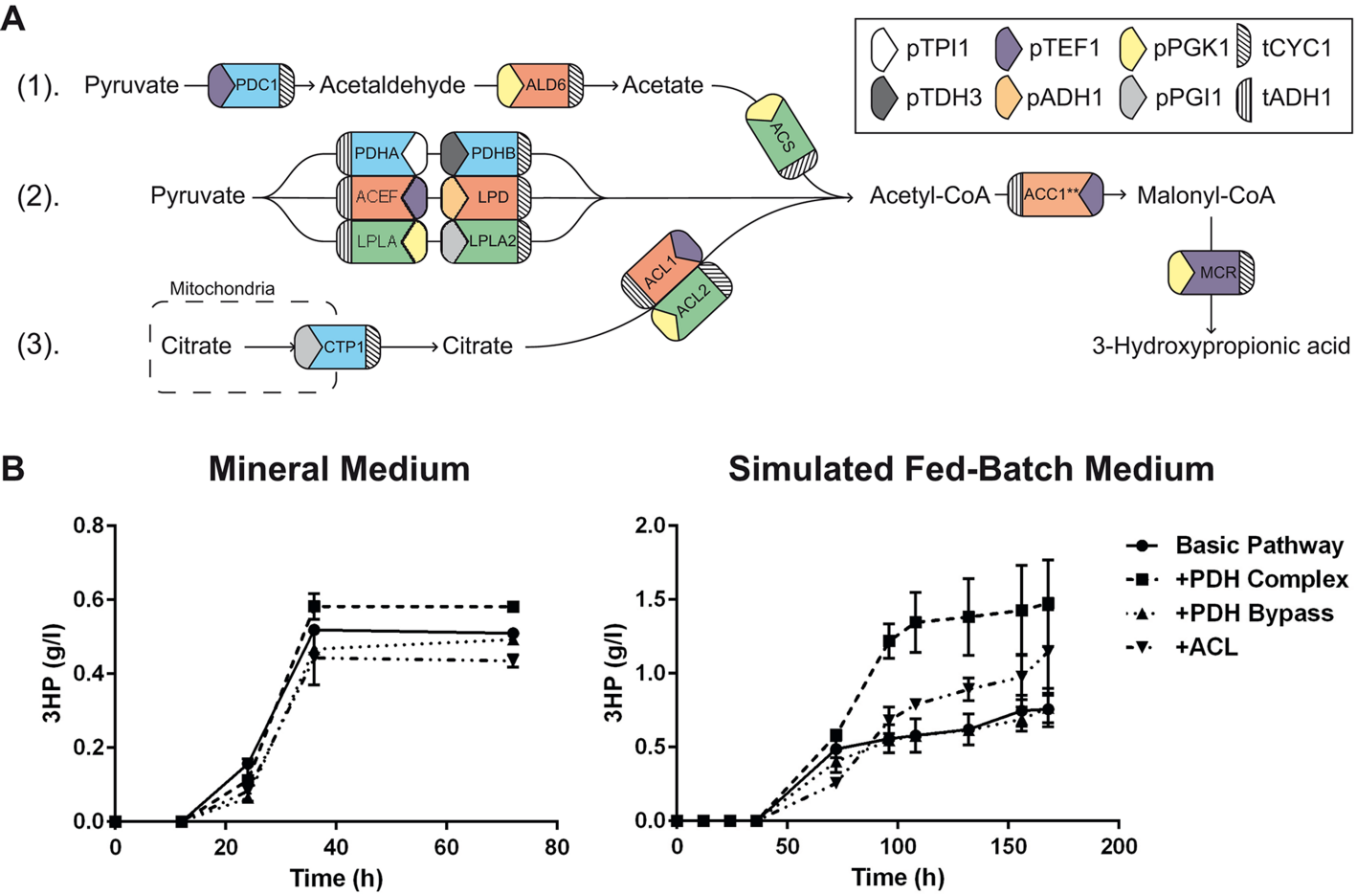
Effect of acetyl‐CoA supply strategies on the production of 3‐hydroxypropionic acid. (**A**) Three different pathways taken to produce acetyl‐CoA in the cytosol; 1. The PDH bypass, 2. The PDH complex of *E. faecalis*, 3. The ATP‐dependent citrate lyase strategy from *Y. lipolytica*. Colored arrows denote which promoter was used, and the direction of the arrow indicates in which direction the gene is transcribed. A left facing arrow signifies that the gene is translated on the anti‐sense strand and vice versa. The box at the end of the gene represents which terminator sequence is used. (**B**) Production of 3‐hydroxypropionic acid over time in the engineered laboratory strain in mineral or simulated fed‐batch medium. The cultivations were carried out in triplicate, the error bars show standard deviation.

As a test case, a pathway to produce 3HP was inserted into two different *S. cerevisiae* strains, CEN.PK113‐7D and Ethanol Red, and the strains were further engineered for improved acetyl‐CoA supply. The basic pathway strains were constructed by inserting a modified acetyl‐CoA carboxylase *ACC1* ^S659A,S1157A^ and a malonyl‐CoA reductase *MCR* from *Chloroflexus aurantiacus.* Three different strategies to boost acetyl‐CoA production in the cytosol were compared (Fig. 3A). One of the strategies relied on expression of a functional bacterial pyruvate dehydrogenase complex from *Enterococcus faecalis* in the cytoplasm, which required expression of genes encoding subunits E1α, E1β, E2, and E3 of PDH complex, as well as two genes involved in lipoylation of E2 30. Another strategy was based on overexpression of the pyruvate dehydrogenase bypass, namely pyruvate decarboxylase *PDC1*, aldehyde dehydrogenase *ALD6* and acetyl‐CoA synthase from *Salmonella enterica* *ACS*_*SE*_ 31. The third strategy encompassed overexpression of ATP‐dependent citrate lyase, consisting of two subunits *ACL1* and *ACL2*, and a mitochondrial citrate transporter protein from *Y. lipolytica*. The genes required for different strategies were cloned into three EasyClone‐MarkerFree plasmids and transformed into yeast strains expressing basic 3HP pathway along with the triple gRNA helper vector (pCfB3052 for CEN.PK and pCfB4668 for Ethanol Red).

Overexpression of the cytoplasmic PDH complex proved to be the most successful strategy in CEN.PK, with an improvement in 3HP final titer of 19% over the basic strain in mineral medium, and 95% in the simulated fed‐batch medium (Fig. 3). The PDH bypass showed no improvement. The ACL strategy performed worse than the basic pathway in MM, but interestingly showed an improvement of 60% in FIT (Fig. 3B). Growth profiles can be found in Supporting information, Fig. S1.

In the diploid industrial strain (Ethanol Red), the PDH complex also performed well, with an improvement of 23% over the basic strain (Supporting information, Fig. S2). These results confirm that the bacterial PDH complex from *Enterococcus faecalis* is a robust and efficient way to improve cytosolic acetyl‐CoA levels in yeast. We confirmed that EasyClone‐MarkerFree method integrates the expression cassettes on both homologous chromosomes, when applied in diploid strains. We performed qPCR on the diploid industrial strain (Ethanol Red) carrying the genes for the PDH complex. All of the six genes were shown to have a copy number of 2. The PDH bypass seems to have performed poorly, contradicting previous works. This may be due to the low activity of the 3HP biosynthetic genes in this work, as we only integrated *ACC1* and *MCR* in single copies. In other studies, where we expressed *ACC1* and *MCR* from either a high‐copy episomal vector or integrated them in multiple copies into the genome, the overexpression of PDH bypass did have a positive effect on the 3HP production 32, 33, 34.

## 4 Concluding remarks

The EasyClone‐MarkerFree vector toolkit can be used to simultaneously introduce one to three integration cassettes into the genome of *S. cerevisiae* without the use of selection markers. Using standardized BioBricks, the integration cassettes can be constructed for overexpression of one or two genes per integration site. In this study we successfully integrated up to six genes in a single transformation with 60–70% targeting efficiency. The system is well suited for strain construction via multiple iterative metabolic engineering cycles. We have shown that it performs well in both the haploid laboratory CEN.PK strain, and also in the diploid industrial Ethanol Red strain. The EasyClone‐MarkerFree vector toolkit can be obtained from AddGene.

## Supporting information

As a service to our authors and readers, this journal provides supporting information supplied by the authors. Such materials are peer reviewed and may be re‐organized for online delivery, but are not copy‐edited or typeset. Technical support issues arising from supporting information (other than missing files) should be addressed to the authors.

Supporting Information
