# Supplementary material for: EasyClone‐MarkerFree: A vector toolkit for marker‐less integration of genes into Saccharomyces cerevisiae via CRISPR‐Cas9
Source: Biotechnol J. 2016 Jun 23;11(8):1110–7. doi: 10.1002/biot.201600147 (PMC5094547; doi:10.1002/biot.201600147)
Supplement: Supplementary file 1 — Supporting Information [file BIOT-11-1110-s001.zip › biot201600147-sup-0001-suppinfo.pdf]

Biotechnology
Journal

Supporting Information for DOI 10.1002/biot.201600147

EasyClone-MarkerFree: A vector toolkit for marker-less integration of genes into *Saccharomyces cerevisiae* via CRISPR-Cas9

Mathew M Jessop-Fabre, Tadas Jakočiūnas, Vratislav Stovicek, Zongjie Dai, Michael K Jensen, Jay D Keasling, Irina Borodina

© 2016 Wiley-VCH Verlag GmbH & Co. KGaA, Weinheim

Supplementary Materials

Table S1. List of primers used in this study

ID	NAME	SEQUENCE (5'->3')	Application
Primers used for construction of EasyClone-MarkerFree plasmids and gRNA helper plasmids			
401	ID401pIntFwdU	ACCCAAUTCGCCCTATAGTGAGTCG	For amplification of the EasyClone vectors minus the markers
402	ID402pintRevU	ACGCGAUCTTCGAGCGTCCCAAAACC	For amplification of the EasyClone vectors minus the markers
10277	gRNArev	GATCATTTATCTTTCACTGCGGAGAAG	universal primer for amplification of gRNA cassette carrying plasmid
11150	X-2_gRNAerFW	CTCTCGAAGTGGTCACGTGTGTTTTAGAGCTAGAA	For amplification of X-2 targeting gRNA helper vector for ethanol red, used with 10277
11151	X-4_gRNAerFW	CGCCATTCAAGAGTAGCAACGTTTTAGAGCTAGAA	For amplification of X-2 targeting gRNA helper vector for ethanol red, used with 10277
11152	XI-2_gRNAerFW	TTGATCAGTTGATCAGTTGAGTTTTAGAGCTAGAA	For amplification of X-2 targeting gRNA helper vector for ethanol red, used with 10277
11153	XI-5_gRNAerFW	TGAGAATAATGTTGTAAAACGTTTTAGAGCTAGAA	For amplification of X-2 targeting gRNA helper vector for ethanol red, used with 10277
10525	TJOS-62 (P1F)	CGTGCGAUAGGGAACAAAAGCTGGAGCT	Amplify 1st gRNA expression sequence for triple gRNA vectors
10526	TJOS-63 (P2F)	AGTGCAGGUAGGGAACAAAAGCTGGAGCT	Amplify 2nd gRNA expression sequence for triple gRNA vectors
10527	TJOS-64 (P3F)	ATCTGTCAUAGGGAACAAAAGCTGGAGCT	Amplify 3rd gRNA expression sequence for triple gRNA vectors
10529	TJOS-65 (P1R)	CACGCGAUTAACTAATTACATGACTCGA	Amplify 3rd gRNA expression sequence for triple gRNA vectors
10530	TJOS-66 (P2R)	ACCTGCACUTAACTAATTACATGACTCGA	Amplify 1st gRNA expression sequence for triple gRNA vectors
10531	TJOS-67 (P3R)	ATGACAGAUTAACTAATTACATGACTCGA	Amplify 2nd gRNA expression sequence for triple gRNA vectors
Primers used for PAM site removal by site-directed mutagenesis			
11399	TJOS-120	ATTAATGCCTCAGCACTAGT	Universal primer for PAM site removal in EasyClone vectors
11354	TJOS-89F	CCACTTTTCAATGAAACGGA	Primer to remove PAM site in pCfB2900
11395	TJOS-116	ACATGGGAAGATTCGCTTTT	Primer to remove PAM site in pCfB2901
11396	TJOS-117	AGTTTCTTGGCATTGGCAAT	Primer to remove PAM site in pCfB2902
11397	TJOS-118	CTATTGGCTGCTTCATAGTA	Primer to remove PAM site in pCfB2905
11398	TJOS-119	AGTTTACTCAATTCTTGAAG	Primer to remove PAM site in pCfB2908
11400	TJOS-121	CGTGAATCAACTGCACATAC	Primer to remove PAM site in pCfB2906
11355	TJOS-90F	CGACTCTCTCGAAATTTTTC	Primer to remove PAM site in pCfB2907
Primers used for amplification of gene and promoter BioBricks			
6	PTEF1_rv	CACGCGAUGCACACACCATAGCTTC	For amplification of the TEF1 promoter
7	PPGK1_fw	CGTGCGAUGGAAGTACCTTCAAAGA	For amplification of the PGK1 promoter
53	ACC1m_fw	CGT GCG AUT CAT TTC AAA GTC TTC AAC AAT TT	For amplification of ACC1** gene

177	CaMCR_rv_NEW	CACGCGAUTCAGACTGTAATGGCTCTACCTC	For amplification of MCR gene
312	GFPopt_rv	CACGCGAU TCA TTTGTAGAGCTCATCCATGC	For amplification of GFP gene
645	ACSse_U1_rv (ID645)	CGTGCGAUTCATGATGGCATAGCAATAG	For amplification of ACS gene
739	ald6_U2_rv (ID739)	CACGCGAUTCACAACTTAATTCTGACAGCTTTTAC	For amplification of ALD6 gene
842	YlAcly1_U1_rv (ID842)	CGTGCGAU TCA TGATCGAGTCTTGGCCTTG	For amplification of ACL1 gene
844	YlAcly2_U2_rv (ID844)	CACGCGAU TCA AACTCCGAGAGGAGTGGAAG	For amplification of ACL2 gene
846	YlCtp1_U1_rv (ID846)	CGTGCGAU TCA AAGAATCTCCATGATCTTC	For amplification of CTP1 gene
1188	pdc1_U1longer_rv (ID1188)	CGTGCGAUTCATTGCTTAGCGTTGGTAGCAGCAGTC	For amplification of PDC1 gene
1564	PTEF1->_fw (ID1564)	CGTGCGAUGCACACACCATAGCTTC	For amplification of TEF1 promoter
10549	lplA_U2_fw	ATCTGTCAUAAAACAATGAGATACGTTATCATGCAATCC	For amplification of LPLA gene
10550	lplA_U_rv	CACGCGAUTCATTAGTCAACCAACAAGTGGACG	For amplification of LPLA gene
10684	pdhB_U2_fw	ATCTGTCAUAAAACAATGGCACAAAAGACTATGATCCAAG C	For amplification of PDHB gene
10685	pdhB_U_rv	CACGCGAUTCATTAAAATTCAACAATTTCTCTGG	For amplification of PDHB gene
10686	pdhA_U1_fd	AGTGCAGGUAAAACAATGGCAAAAGCAAAGAAGC	For amplification of PDHA gene
10687	pdhA_U_rv	CGTGCGAUTTACTTTGATTCTTTGGCTTCG	For amplification of PDHA gene
10688	aceF_U2_fw	ATCTGTCAUAAAACAATGGCCTATCAATTCAAGTTGCC	For amplification of ACEF gene
10689	aceF_U_rv	CACGCGAUTCAACCTTCCATCAATAA	For amplification of ACEF gene
10690	lpd_U1_fw	AGTGCAGGUAAAACAATGGTTGTTGGTGAC	For amplification of LPD gene
10691	lpd_U_rv	CGTGCGAUTCATTAGATATGTATAGGCAAACC	For amplification of LPD gene
Sequencing primers			
891	XII-1-up-out-sq ID891	CTGGCAAGAGAACCACCAAT	Verifies UP region of chromosome site XII-1 with ID2221
893	XII-2-up-out-sq ID893	CGAAGAAGGCCTGCAATTC	Verifies UP region of chromosome site XII-2 with ID2221
897	XII-4-up-out-sq ID897	GAACTGACGTCGAAGGCTCT	Verifies UP region of chromosome site XII-4 with ID2221
899	XII-5-up-out-sq ID899	CCACCGAAGTTGATTTGCTT	Verifies UP region of chromosome site XII-5 with ID2221
901	X-2-up-out-sq ID901	TGCGACAGAAGAAAGGGAAG	Verifies UP region of chromosome site X-2 with ID2221
903	X-3-up-out-sqID903	TGACGAATCGTTAGGCACAG	Verifies UP region of chromosome site X-3 with ID2221
905	X-4-up-out-sqID905	CTCACAAAGGGACGAATCCT	Verifies UP region of chromosome site X-4 with ID2221
907	Ny-XI-1-up-sqID907	CTTAATGGGTAGTGCTTGACACG	Verifies UP region of chromosome site XI-1 with ID2221
909	XI-2-sq-fwID909	GTTTGTAGTTGGCGGTGGAG	Verifies UP region of chromosome site XI-2 with ID2221
911	XI-3- up-out-sqID911	GTGCTTGATTTGCGTCATTC	Verifies UP region of chromosome site XI-3 with ID2221
2221	JM234_ColoPCR_vec_TADH1_toward s out	GTTGACACTTCTAAATAAGCGAATTTC	Verifies UP regions of chromosome sites
8418	verif ChrXI-5_up (JM269)	CTCAATGATCAAAATCCTGAATGCA	Verifies UP region of chromosome site XI-5
qPCR Primers			
14120	ALG9_qPCR_fw	CCGTTGCCATGTTGTTGTATG	Primer to use as a control for qPCR
14121	ALG9_qPCR_rv	GCCAGGAAATTGTACGCTAAAC	Primer to use as a control for qPCR
15553	(AceF qPCR) FWD	GCGACTTCTGGGTAGTTGATAA	For amplification of a short section of the E. faecalis AceF gene

15554	(AceF qPCR) REV	TAACCGCACAAGATATGAGAGAC	For amplification of a short section of the E. faecalis AceF gene
15555	(lpd qPCR) FWD	GCCTTCACAGACCCTGAATTA	For amplification of a short section of the E. faecalis lpd gene
15556	(lpd qPCR) REV	CTCTACCGTTACCAGCGAATG	For amplification of a short section of the E. faecalis lpd gene
15557	(pdhA qPCR) FWD	GTCGTCACCGGACAAAGTAT	For amplification of a short section of the E. faecalis pdhA gene
15558	(pdhA qPCR) REV	AGCCAAGGAAGCAAGAGATT	For amplification of a short section of the E. faecalis pdhA gene
15559	(lplA qPCR) FWD	CCATGCGTTATCTTGGGTAGAA	For amplification of a short section of the E. faecalis lplA gene
15560	(lplA qPCR) REV	CACCACCACCAGACAATCTT	For amplification of a short section of the E. faecalis lplA gene
15561	(lplA2 qPCR) FWD	GCACCATCAGCAACATTCATC	For amplification of a short section of the E. faecalis lplA2 gene
15562	(lplA2 qPCR) REV	CAGAAACTGGGACTGGAACTAC	For amplification of a short section of the E. faecalis lplA2 gene
15563	(pdhB qPCR) FWD	CAGAGAAGAAGTCCCAGATGAAG	For amplification of a short section of the E. faecalis pdhB gene
15564	(pdhB qPCR) REV	CATGGCACCGTAGGTTATAATAGA	For amplification of a short section of the E. faecalis pdhB gene

Table S2. List of BioBricks used in this study

ID	Name	Primers used	Template	Description
BB8	<-TEF1	5, 6	p0029	TEF1 promoter in position P1
BB157	pTEF1-ACC1_pPGK1-CaMCR	53, 177	p0298	acc1 - double mutant under control of TEF1 promoter in position 1. mcr gene from *Chloroflexus aurantiacus* under control of PGK1 promter in position 2
BB183	ctp1<-	845, 846	*Y. lipolytica* genomic DNA	Mitochondrial citrate transporter from *Y. lipolytica* in position 1
BB867	pTEF1-GFP	1564, 312	p2199	GFP protein under control of TEF1 promoter in position 2
BB903	pTDH3->	10720, 279	p2525	TDH3 promoter in position 2 to fuse to a promoter in position 1
BB904	<-pdhA	10686, 10687	Codon optimised pdhA gene string	pdhA subunit of PDH complex from *E. faecalis* in position 1
BB905	pdhB->	10684, 10685	Codon optimised pdhB gene string	pdhB subunit of PDH complex from *E. faecalis* in position 2
BB906	<-lpd	10690, 10691	Codon optimised lpd gene string	lpd subunit of PDH complex from *E. faecalis* in position 1
BB907	aceF->	10688, 10689	Codon optimised aceF gene string	aceF subunit of PDH complex from *E. faecalis* in position 2
BB908	<-pTEF1	10721, 1750	p0029	TEF1 promoter in position 1 to fuse to a promoter in position 2
BB909	pPGK1->	10722, 293	p0029	PGK1 promoter in position 1 to fuse to a promoter in position 1
BB918	<-pTPI1	10764, 10765	*S. cerevisiae* genomic DNA	TPI1 promoter in position 1 to fuse to a promoter in position 2
BB1021	<-pPGI1	10545, 10546	*S. cerevisiae* genomic DNA	PGI1 promoter in position 1 to fuse to a promoter in position 2
BB1022	lplA->	10549, 10550	Codon optimised pdhA gene string	lplA lipoylation gene of PDH complex from *E. faecalis* in position 1
BB1023	<-lplA2	10547, 10548	Codon optimised pdhA gene string	lplA2 lipoylation gene of PDH complex from *E. faecalis* in position 2
BB1127	ylacly1<-pTEF1	842, 6	p0626	acly2 gene from *Y. lipolytica* under the control of TEF1 promoter in position 1
BB1128	pPGK1->ylacly2	844, 7	p0626	acly1 gene from *Y. lipolytica* under the control of TEF1 promoter in position 2

BB1 449	pdc1<-pTEF1	1188, 6	p0382	pdc1 gene under control of TEF1 promoter in position 1
BB1 450	pPGK1->ALD6	739, 7	p0380	ald6 gene under control of PGK1 promoter in position 2
BB1 451	ACSse<-pTEF1	645, 6	p0380	acs from *S. enterica* under control of TEF1 promoter in position 1

Table S3. List of plasmids used in this study

ID	Name	Description	Addgene reference	Source
	Cas9 expression plasmid			
pCfB2312	TEF1p-Cas9-CYC1t_kanMX	Episomal plasmid for Cas9 expression		[1]
	Intermediate vectors			
pCfB2900	X-3-intermediate for MF	Amplified from plasmid pCfB127, using primers 401 and 402 . Contains PAM site		This study
pCfB2901	X-4-intermediate for MF	Amplified from plasmid pCfB2070, using primers 401 and 402 . Contains PAM site		This study
pCfB2902	XI-1-intermediate for MF	Amplified from plasmid pCfB2328, using primers 401 and 402 . Contains PAM site		This study
pCfB2905	XI-5-intermediate for MF	Amplified from plasmid pCfB387, using primers 401 and 402 . Contains PAM site		This study
pCfB2906	XII-1-intermediate for MF	Amplified from plasmid pCfB2072, using primers 401 and 402 . Contains PAM site		This study
pCfB2907	XII-2-intermediate for MF	Amplified from plasmid pCfB120, using primers 401 and 402 . Contains PAM site		This study
pCfB2908	XII-4-intermediate for MF	Amplified from plasmid pCfB130, using primers 401 and 402 . Contains PAM site		This study
	EasyClone-MarkerFree vectors			
pCfB2899	X-2-MarkerFree backbone	Amplified from plasmid pCfB126 ,using primers 401 and 402.	73271	This study
pCfB2903	XI-2-MarkerFree	Amplified from plasmid pCfB384 ,using primers 401 and 402.	73275	This study
pCfB2904	XI-3-MarkerFree	Amplified from plasmid pCfB2118 ,using primers 401 and 402.	73276	This study
pCfB2909	XII-5-MarkerFree	Amplified from plasmid pCfB2073 ,using primers 401 and 402.	73281	This study
pCfB3034	X-3-MarkerFree	PAM site in pCfB2900 mutated with primers 11399 and 11354	73272	This study
pCfB3035	X-4-MarkerFree	PAM site in pCfB2901 mutated with primers 11399 and 11395	73273	This study
pCfB3036	XI-1-MarkerFree	PAM site in pCfB2902 mutated with primers 11399 and 11396	73274	This study
pCfB3037	XI-5-MarkerFree	PAM site in pCfB2905 mutated with primers 11399 and 11397	73277	This study
pCfB3038	XII-1-MarkerFree	PAM site in pCfB2906 mutated with primers 11399 and 11400	73278	This study
pCfB3039	XII-2-MarkerFree	PAM site in pCfB2907 mutated with primers 11399 and 11355	73279	This study
pCfB3040	XII-4-MarkerFree	PAM site in pCfB2908 mutated with primers 11399 and 11398	73280	This study
	Single target gRNA helper vectors			
pCfB3020	gRNA-X-2	gRNA sequence for targetting site X-2 USER cloned into pCfB2926	73282	This study
pCfB3041	gRNAX-3	gRNA sequence for targetting site X-3 USER cloned into pCfB2926	73283	This study
pCfB3042	gRNAX-4	gRNA sequence for targetting site X-4 USER cloned into pCfB2926	73284	This study
pCfB3043	gRNAXI-1	gRNA sequence for targetting site XI-1 USER cloned into pCfB2926	73285	This study
pCfB3044	gRNAXI-2	gRNA sequence for targetting site XI-2 USER cloned into pCfB2926	73286	This study
pCfB3045	gRNAXI-3	gRNA sequence for targetting site XI-3 USER cloned into pCfB2926	73287	This study
pCfB3046	gRNAXI-5	gRNA sequence for targetting site XI-5 USER cloned into pCfB2926	73288	This study

pCfB3047	gRNAXII-1	gRNA sequence for targetting site XII-1 USER cloned into pCfB2926	73289	This study
pCfB3048	gRNAXII-2	gRNA sequence for targetting site XII-2 USER cloned into pCfB2926	73290	This study
pCfB3049	gRNAXII-4	gRNA sequence for targetting site XII-4 USER cloned into pCfB2926	73291	This study
pCfB3050	gRNAXII-5	gRNA sequence for targetting site XII-5 USER cloned into pCfB2926	73292	This study
pCfB3588	EthanolRed gRNA X-2	Amplified with primers 10277 and 11150 , using pCfB3041 as template		This study
pCfB3589	EthanolRed gRNA X-4	Amplified with primers 10277 and 11151, using pCfB3041 as template		This study
pCfB3590	EthanolRed gRNA XI-2	Amplified with primers 10277 and 11152 , using pCfB3041 as template		This study
pCfB3591	EthanolRed gRNA XI-5	Amplified with primers 10277 and 11153 , using pCfB3041 as template		This study
	Triple target gRNA helper vectors			
pCfB3051	gRNAX-3XI-2XII-2	Three different target site cassettes amplified using primers 10525 and 10530, 10526 and 10531, 10527 and 10529 . Sections are amplified from the respective single gRNAs	73293	This study
pCfB3052	gRNAX-4XI-3XII-5	Three different target site cassettes amplified using primers 10525 and 10530, 10526 and 10531, 10527 and 10529 . Sections are amplified from the respective single gRNAs	73294	This study
pCfB3053	gRNAX-2XI-5XII-4	Three different target site cassettes amplified using primers 10525 and 10530, 10526 and 10531, 10527 and 10529 . Sections are amplified from the respective single gRNAs	73295	This study
pCfB4668	Ethanol Red p-gRNA X-4 XI-3 XII-5	Three different target site cassettes amplified using primers 10525 and 10530, 10526 and 10531, 10527 and 10529 . Sections are amplified from the respective single gRNAs		This study
	GFP Vectors			
pCfB3008	X-2-MarkerFree-GFP	Biobrick 867, USER cloned into plasmid pCfB2899		This study
pCfB3009	X-3-MarkerFree-GFP	Biobrick 867, USER cloned into plasmid pCfB3034		This study
pCfB3010	X-4-MarkerFree-GFP	Biobrick 867, USER cloned into plasmid pCfB3035		This study
pCfB3011	XI-1-MarkerFree-GFP	Biobrick 867, USER cloned into plasmid pCfB3036		This study
pCfB3012	XI-2-MarkerFree-GFP	Biobrick 867, USER cloned into plasmid pCfB2903		This study
pCfB3013	XI-3-MarkerFree-GFP	Biobrick 867, USER cloned into plasmid pCfB2904		This study
pCfB3014	XI-5-MarkerFree-GFP	Biobrick 867, USER cloned into plasmid pCfB3037		This study
pCfB3015	XII-1-MarkerFree-GFP	Biobrick 867, USER cloned into plasmid pCfB3038		This study
pCfB3016	XII-2-MarkerFree-GFP	Biobrick 867, USER cloned into plasmid pCfB3039		This study
pCfB3017	XII-4-MarkerFree-GFP	Biobrick 867, USER cloned into plasmid pCfB3040		This study
pCfB3018	XII-5-MarkerFree-GFP	Biobrick 867, USER cloned into plasmid pCfB2909		This study
	Production vectors			
pCfB3069	X-3-MF-MCR-ACC1	Biobrick 157, USER cloned into plasmid pCfB3034		This study
pCfB3472	pdhA-pdhB	Biobricks 904, 905, 903, 918, USER cloned into plasmid pCfB3035		This study
pCfB3473	aceF-lpd	Biobricks 906, 907, 908, 909, USER cloned into plasmid pCfB2904		This study
pCfB3479	lplA2-lplAXII-5	Biobricks 1022, 1023, 1021, 909, USER cloned into plasmid pCfB2909		This study
pCfB3584	X-4 ylacly1<-pTEF1	Biobrick 1127, USER cloned into plasmid pCfB3035		This study
pCfB3585	XI-3 pPGK1->ylacly2	Biobrick 1128, USER cloned into plasmid pCfB2904		This study
pCfB3587	pdc X-4	Biobrick 1449, USER cloned into plasmid pCfB3035		This study
pCfB3592	pPGK1->ALD6	Biobrick 1450, USER cloned into plasmid pCfB2904		This study
pCfB3593	ACS-pPGK1 XII-5	Biobrick 1451, USER cloned into plasmid pCfB2909		This study

pCfB3594	ylCtp1<-pTEF1 XII-5	Biobricks 183, 8 USER cloned into plasmid pCfB2909		This study

Table S4. Chromosomal coordinates of the EasyClone-MarkerFree integration sites

Integration site or Gene name	Chromosomal coordinates according to Saccharomyces genome database (http://www.yeastgenome.org/).
X-2	Chr X: 194944..195980
X-3	Chr X: 223616..224744
X-4	Chr X: 236336..237310
XI-1	Chr XI: 67491..68573
XI-2	Chr XI: 91575..92913
XI-3	Chr XI: 93378..94567
XI-5	Chr XI: 11779..118967
XII-1	Chr XII: 795787..796720
XII-2	Chr XII: 808805..809939
XII-4	Chr XII:830227..831248
XII-5	Chr XII: 839226..840357

Table S5. Sequences of *E. faecalis* PDH complex genes

Gene	Sequence (5'->3')
pdhA	atggcaaaagcaaagaagcaaaaacctatagatttcaaagaattgatggctaaagtagacgcagatttccctacattccaaatattagatcaagacggt<br>aaaatagttaacgaagatttggtcccagatttgtctgacgaagaattggttgaattgatgacaagaatggtatggtctagagttttggatcaaagatcaac<br>cgcattgaacagacaaggtagattaggtttctttgctccaaccgcaggtcaagaagcatcccaattagccagtcaattcgctatggaaaaggaagattat<br>ttgttgccaggttacagagacgtccctcaattggtacaacatggtttgcctttaagagaagctttcttatggtcaagaggtcacgttgctggtaattattacg<br>cagaagatttgaacgccttaccacctcaaattataatcggtgcccaatatattcaagctgcaggtgtcgctttgggtttgaagaaaagaggtaaagaaaa<br>cgttgtctttacttacacaggtgacggtggttcttcacagggtgacttctacgaagcaattaatttcgccggtgcttaccaagctaacggtgtttttattatcc<br>aaaacaacggtttcgctatatccacaccaagagaaaaacaaacagccgctaagaccttggcccaaaaagctgttgcagccggtataccaggtatccaa<br>gtagatggtatggaccctttagctgtttatgcaatagccaaggaagcaagagattggagtgctgctggtaatggtccagtcttgatcgaaaccttaacttat<br>agatacggtccacatactttgtccggtgacgaccctacaagatatcgtagtaaggaaatggatgacgaatgggttcaaaaagatcctttaaccagattca<br>gaaagtacttgactgataaaggtttatggtccgaagcaaaggaagaagaaatcatcgaaaagactaaggaagaaattaaggttgctatagcagaagcc

	gataaagctccaaaacaaaaggtctctgactttttgaagaacatgttcgaagtacaacctcaaacaatcaaagaacaaatagcattctacgaagccaaa<br>gaatcaaagtaa
pdhB	atggcacaaaagactatgatccaagctataactgacgccttagccttagaattagaaaaagatgaaaatgtattgatattcggtgaagatgtaggtaata<br>acggtggtgtttttagagccacagaaggtttgcaagaaaagtttggtgaagatagagttttcgacaccccattggctgaatccggtataggtggtttagctt<br>ttggtttggcattacaaggttatagaccagttcctgaaatccaatttttcggttttgtcttcgaagtattcgatgaaattgtcggtcaaatggctagaactag<br>atacagaatgggtggtaccagaaatatgccaattactgtaagagcaccttttggtggtggtgttcatacaccagaattacactctgataacttggaaggttt<br>aatagcacaatcccctggtgttagagttgtcatcccaagtaatccttatgatgccaagggtttgttgatctcttcaatcagatccaacgacccagtagtttat<br>ttggaacatatgaagttgtacagaagtttcagagaagaagtcccagatgaagcatatgaagtacctttggacaaagctgcagttactagagaaggtaca<br>gatgtctctattataacctacggtgccatggttagagaagctattaaagccgctgattctttagcaaaggacaatatatcagccgaaatcattgatttgaga<br>acagttgctcctttagacgtcgaaaccataatcaactcagttgaaaagactggtagagtcgtagttgtccaagaagcccaaaagcaagctggtgttggtg<br>caatggtagtttccgaaattagtgaaagagctgtcttgtctttagaagcaccaataggtagagtatcagcaccagatactatctttcctttcggtcaagccg<br>aaaatatttggttgcctaacgctaaagacatcgaagcaaaggccagagaaattgttgaattttaa
lpd	atggttgttggtgactttgctattgaattggatacagtagttattggtgccggtcctggtggttatgttgccgccatcagagccgcagaaatgggtcaaaag<br>gttgctatcatcgaaagagaatacatcggtggtgtttgtttgaacgtcggttgcataccatcaaaagctttgatcgctgcaggtcatcactaccaagaagc<br>acaagattcttcaacttttggtgtcacagcaaagggtgtaaaattagacttcgccaagacccaagattggaaagacaataccgttgtcaagtctttgactt<br>caggtgtaggcatgttgttgaagaaacataaggttgaaatcatcgaaggtgaagcctttttcgtagatgaaaacacattgagagttattcacccagactct<br>gcacaaacttactcttttaataacgcaatcgttgccacaggttctagaccaatcgaaattcctggttttaagttcggtggtagagttttggattcaactggtg<br>gtttgaatttgaaggaagttcctaaaaagttcgtcataatcggtggtggtgttattggtgctgaattaggtggtgcttatgcaaacttgggttctgaagtcac<br>aatcttagaaggttccccaagtattttgcctacctacgaaaaggatatggttaaagtagttactgatgacttcaaaaagaaaaatgtaaccattgttacttc<br>tgccatggctaaagaagctgttgataatggtgactcagtcactgtaaagtatgaagtcaacggtaaagaagaatctgtagaagctgattacgttatggtc<br>acagtaggtagaagaccaaataccgatgacttgggtttagaacaagcaggtgttgaaattggtgaaagaggtttaataccagttgataaccaaggtaga<br>acaaacgtcaaaaacatcttcgctatcggtgacatcgttcctggtgccgctttggctcataaggcatcctatgaagccaaaatcgcagccgaagctattag<br>tggtaaaaaggttgcagtcgattacaaggccatgccagcagttgccttcacagaccctgaattagcatccgttggtatgaccgttgctgaagcaaaagaa<br>gctggtattgaagcaaagggttacaaatttccattcgctggtaacggtagagcaatctcattggataagactgaaggttttatgagattggtcactacagt<br>agaagacaacgtcattataggtgcccaaattgctggtgtaggtgcttccgatatgataagtgaattggccttagctatcgaatccggtatgaatgccgaag<br>atattgctttaacaatacatccacaccctagtttgggtgaaattacaatggataccgcagaattggccttaggtttgcctatacatatctaa
aceF	atggcctatcaattcaagttgcctgacatcggtgaaggtatcgcagaaggtgaaatcgtaaaatggtttgtaaagcctggtgacacaatcaacgaagatg

	acactttgttggaagttcaaaacgataagtctgtcgaagaaataccatcacctgtaaccggtactgttaaaaatatcgttgtccctgaaggtacagtcgct<br>aacgtaggtgacgttttgattgaaatagacgcaccaggtcacgaagataatgacgctgcaccagccgctcctgcacaagaacaaactccagcccaacct<br>gcagccgttcctactacagaagctgcaggtggtttctttcaattcaaattgccagatataggtgaaggtatagctgaaggtgaaattgtcaaatggttcgta<br>aaggcaggtgacactataaatgaagatgactctttgttggaagtacaaaacgataagtccgttgaagaaatcccaagtcctgttacaggtaccgtcaaaa<br>atattgtagttccagaaggtactgttgccaatgttggtgacgttttggtcgaaattgacgcaccaggtcataattctgccgctccagcagccgctgcacctg<br>ctactgatgcaccaaaagccgaagcttccgcaccagccgctagtacaggtgtcgtagcagccgctgatcctaacaagagagtcttagcaatgccatcagt<br>tagacaatatgccagagaaaaagatgtagacattacacaagttactgctactggtaaaggtggtagagttatcaaggccgatattgacgcttttgtttccg<br>gtggtagtcaagcagcccctgctactgaagctgcagccacagaagctgcaccaaaagctgaagccgctgcaccaaaagccgctcctaaggcattcacat<br>ccgatttgggtgaaatggaaaccagagaaaagatgacccctactagaaaagccattgctaaggcaatggttaattccaaacataccgctccacacgtta<br>ctttgcatgatgaagtagaagttagtaagttgtgggatcacagaaagaagtttaaagacgttgcagccgctaatggtacaaaattgaccttcttaccttat<br>gttgtcaaagctttgacatctaccgttcaaaagttcccaatcttgaacgcatcaatagatgacgcagcccaagaaatagtttacaagaactacttcaacat<br>cggtattgctactgatacagaccatggtttgtacgttccaaacgtcaaaaatgctaacacaaagtcaatgtttgccatagctgatgaaatcaacgaaaaa<br>gctgcattggctattgagggtaaattaaccgcacaagatatgagagacggtaccataactatctctaatataggttcagttggtggtggttggttcacccct<br>gttatcaactacccagaagtcgctatcttaggtgtaggtactattgcccaagaaccagtagttaatgctgatggtgaaattgtcgtaggtagaatgatgaa<br>gttgtctttgtcattcgatcatagaatcgttgacggtgcaactgcccaaaaagctatgaacaacattaaaagattattggcagacccagaattattattgat<br>ggaaggttga
lplA	atgagatacgtcatcatgcaatccagagacatcagagaaaatttggctaccgaagattacttgttgaacaccttgtctttcgaagaaccattggtcttgttc<br>tacattcaagaaccatgcgttatcttgggtagaaatcaaaacgcctacgaagaaattgatttggcttacgctagagaaaagggtatcgttatcactagaa<br>gattgtctggtggtggtgctgtttatgatgatttgggtaatgtctccttctcattcgttgttcaagaaggtcatcaagctttcggtgattttaaggctttcacca<br>agccaattattgaagccttgcataagatgggtgctactggtgctgaaatttctggtagaaacgatttgttgatcgacggtaaaaagttctctggtaatgcta<br>tgtacaccaaaaagggtaagatgtacactcacggtactttgatgtacgatgttgacttggctgaagttcaaagagttttgaccgtttccaagaagaagatc<br>gaatctaagggtactaagtccgttagaggtagagttacaaacttaagaccatacttggacggtaaataccaacaattgaccatcgaagaattcagaaac<br>agattattgatggaattattcgatgtcgaatccttgaccgaaatcgccgaaaaagaatacgttttgactaagaccgaccaacaagaaatcagaaagttgg<br>ttgctgaagtctacggtaatgaagcttggatttttggtgaagctccaaagttcaccatcaagaaagaagaaaagttcaagggtggtatcgttgatgctaga<br>ttgactgttgaaaaaggtaaaatcatcgaattgactatctacggtgattacttcgctaagaaagaaaccgctgaaatagttgctgctttgttgggtgttgat<br>taccaatactcttctatttggcaagctttggctgctttcaacttcgaagattatttcgtcaacatcaccaaagaagaattcgtccacttgttggttgactaa
lplA-2	gtcatcttcgtcccaaacgaaaacaacgatccaagagttaacttggccattgaaacttacttgttgaccgaaatgccattggatgaacctatcttgttgttct

	acatcaacgaaccatccatcatcatcggtagaaatcaaaacaccatcgaagaaatcaacaaagaatacgttgacgaacacggtatccacgttgttagaa<br>gattgtctggtggtggtgctgtttatcatgatcacggtaatttgaacttctcattcatcatgccagatgacggtaactcttttagagatttcgctaaggttacc<br>caacctattattcaagccttgcatgatttgggtgttgaaggtgctgaattgaagggtagaaatgatttggttatcaacgacatgaagttctccggtaatgct<br>atgtatgctaccaatggtagaatgttcgctcatggtactttgatgttcgattccgatatcgatgaagttgtcaacaccttgaaagtcagaaaggacaagatt<br>gaatccaagggtatcaagtccgttagatctagagttaccaacatcaagccattcttgtccgaagataagcaagaaatgactaccgaagaattcagacaa<br>gaaatcttgttgaagattttcggtgtcgactccatcgatcaagttaagacttatgaattgaccgatcaagattgggctgccattaacaaaatctccgaacaa<br>tattacagaaactgggactggaactacggtaaatctccagcttttaacttggaaagaagacacagattcccaatcggttccattgaaatgaagatgaatg<br>ttgctgatggtgccattcaagaaatcaagatcttcggtgatttttttcggtttgggtgaaatcaaggatgtcgaagatattttgaccggtgttaagtacgataa<br>ggcctctttggaagaagccattgatcaaatcgatgtcaagaagtactttggtaacatcgaaaaagaagatttgttgggtttgatctat

Figure S1. Growth profile of the 3-HP-producing laboratory strains in mineral medium (i), and in simulated fed-batch medium (ii).

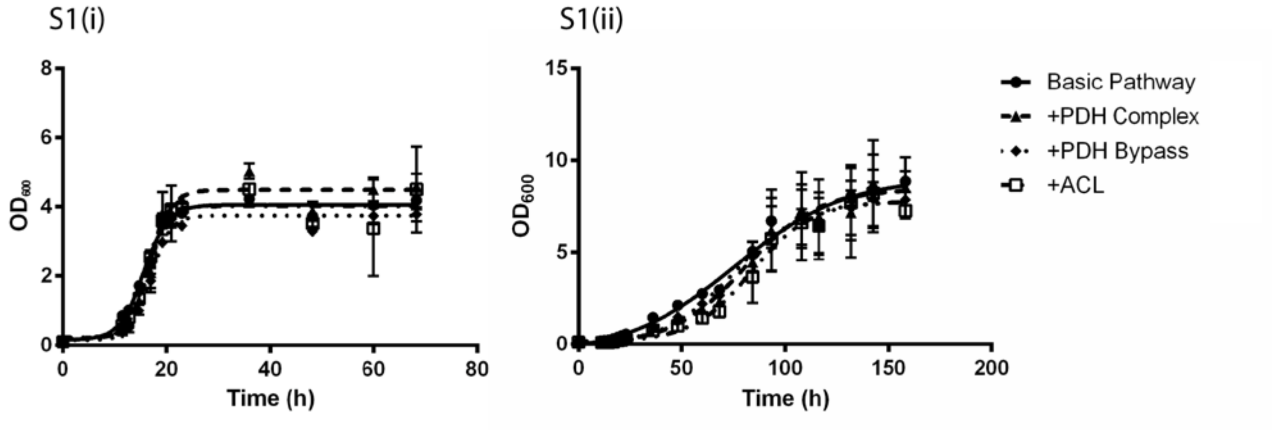

Figure S2. Final titres of 3-hydroxypropionic acid in the engineered industrial Ethanol Red strains.

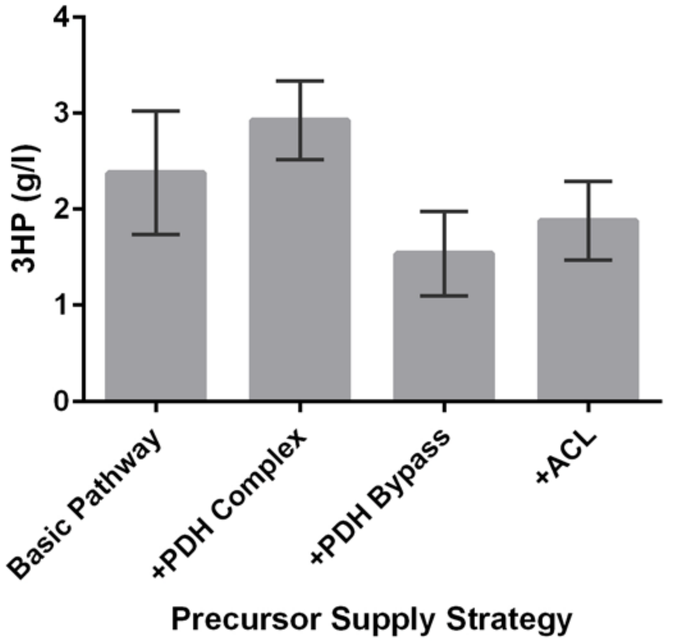

References

[1] Stovicek, V., Borodina, I., Forster, J., CRISPR–Cas system enables fast and simple genome editing of industrial *Saccharomyces cerevisiae* strains. *Metab. Eng. Commun.* 2015, *2*, 13-22.

[2] Mikkelsen, M. D., Buron, L. D., Salomonsen, B., Olsen, C. E. et al., Microbial production of indolylglucosinolate through engineering of a multi-gene pathway in a versatile yeast expression platform. *Metab. Eng.* 2012, *14*, 104-111.
