# Supplementary material for: EasyClone‐MarkerFree: A vector toolkit for marker‐less integration of genes into Saccharomyces cerevisiae via CRISPR‐Cas9
Source: Biotechnol J. 2016 Jun 23;11(8):1110–7. doi: 10.1002/biot.201600147 (PMC5094547; doi:10.1002/biot.201600147)
Supplement: Supplementary file 1 — Supporting Information [file BIOT-11-1110-s001.zip › EasyClone-MarkerFree manual.pdf]

The Novo Nordisk Foundation
Center for Biosustainability

EASYCLONE-MARKERFREE

Marker-less integrative vector set for *Saccharomyces cerevisiae* using CRISPR-Cas9

USER MANUAL

Table of Contents

INTRODUCTION 2
LIST OF EASYCLONE-MARKERFREE VECTORS 3
METHOD OVERVIEW 5
PROTOCOLS 7
Preparation of vectors for USER cloning 7
Primer design 8
Preparation of DNA BioBricks 9
USER cloning of BioBricks into vector 10
Verification of expression vector assembly by colony PCR 11
Linearization of expression vector 12
Transformation of expression vector(s) into yeast (according to Gietz and Woods, 2002) 13
APPENDIX 15
Verification of correct vector integration in the genome by yeast colony PCR 15
Media recipes 17
REFERENCES 19

INTRODUCTION

**Overview**	The collection contains a set of 11 integrative vectors suitable for (over-)expression of (heterologous) genes in both laboratory and industrial *S. cerevisiae* strains, along with 14 gRNA helper vectors that guide Cas9 to the correct cutting site (11 for single site targeting, and 3 for triple site targeting), and a vector encoding the Cas9 protein itself which introduces a double stranded break at a target specified by the gRNA helper vectors. The vectors allow for marker-less insertions of genes into the chromosome through the use of CRISPR-Cas9 technology. The EasyClone system provides a possibility of cloning of up to two genes per vector, when a bidirectional promoter (any of choice) is used, and integration of one to three vectors in one transformation into well-defined 11 yeast chromosomal locations. The biobricks are assembled and cloned into the vectors via Uracil-Specific Excision Reaction (USER) cloning.
**References**	EasyClone-MarkerFree is described in (Jessop-Fabre *et al.,* submitted).<br>EasyClone with detailed description of the cloning procedure (Jensen *et al.*, 2014).<br>Original paper on characterization of integration sites (Mikkelsen *et al.*, 2012).<br>USER cloning method https://www.neb.com/applications/cloning-and-synthetic-biology/user-cloning.
**Related materials**	EasyClone 2.0 is described in (Stovicek *et al.*, 2015a).<br>EasyCloneMulti, a vector set for integration in multiple copies into retrotransposon sites.<br>Vectors for single gene deletions (Stovicek *et al.*, 2015b) via CRISPR-Cas9.<br>Vectors for multiple gene deletions (Jakočiūnas *et al.*, 2015) via CRISPR-Cas9.

LIST OF EASYCLONE-MARKERFREE VECTORS

**Vector characteristics**	The EasyClone-MarkerFree vector set contains 11 integrative vectors with 14 gRNA helper vectors (11 for single site targeting, and 3 for triple site targeting) and 1 vector for the expression of the Cas9 protein.<br>The vectors are designed for integration into 11 specific loci on chromosomes X, XI and XII. The integration sites were chosen for high expression level of the inserted gene and for no interference with the cellular growth (Mikkelsen et al., 2012).<br>The vectors contain USER site, flanked by ADH1 and CYC1 terminators, for cloning of genes and promoters. The cloning can be conveniently accomplished by USER cloning, however other methods can be used as well, such as in-fusion, Gibson, MoClo, etc.<br>The vectors have *E. coli* origin of replication and ampicillin resistance for propagation in *E. coli*.
**Storage**	Upon receipt of the EasyClone-MarkerFree vector set, the plasmids must be transformed into *E. coli* and the transformants selected on LB-amp agar plates. Single colonies should be inoculated in liquid LB-amp overnight and the plasmids extracted from the cells, following the protocol outlined by plasmid extraction kit of choice.

ID	Target Site(s)	Chromosomal coordinates according to Saccharomyces genome database (http://www.yeastgenome.org/).
Cas9 Expression Vector		
pCfB2312	n/a	
EasyClone-MarkerFree Integrative Vectors		
pCfB2899	X-2	Chr X: 194944..195980
pCfB3034	X-3	Chr X: 223616..224744
pCfB3035	X-4	Chr X: 236336..237310
pCfB3036	XI-1	Chr XI: 67491..68573
pCfB2903	XI-2	Chr XI: 91575..92913
pCfB2904	XI-3	Chr XI: 93378..94567
pCfB3037	XI-5	Chr XI: 11779..118967
pCfB3038	XII-1	Chr XII: 795787..796720
pCfB3039	XII-2	Chr XII: 808805..809939
pCfB3040	XII-4	Chr XII:830227..831248
pCfB2909	XII-5	Chr XII: 839226..840357
gRNA Helper Vectors		
pCfB3020	X-2	
pCfB3041	X-3	
pCfB3042	X-4	
pCfB3043	XI-1	
pCfB3044	XI-2	
pCfB3045	XI-3	
pCfB3046	XI-5	
pCfB3047	XII-1	
pCfB3048	XII-2	
pCfB3049	XII-4	
pCfB3050	XII-5	
pCfB3051	X-3, XI-2, XII-2	
pCfB3052	X-4, XI-3, XII-5	
pCfB3053	X-2, XI-5, XII-4	

METHOD OVERVIEW

**Selection of vectors**	For the transformation into specific sites, it is necessary to select a gRNA helper vector that is complementary to the integrative vector(s). Both must target the same site(s).<br>Once a particular integration site is occupied, the strain cannot be transformed with another expression vector targeting the same site.<br>To obtain maximum targeting efficiency the vectors for simultaneous transformations should preferentially be chosen in such a way that they integrate into different chromosomes.<br>When using diploid strains, it can be expected that the expression vectors will integrate on both allelic chromosomes, resulting in two copies per genome.
**Cloning**	The vectors are well suited for cloning of one or two genes into the USER cloning site. The genes can be either native or heterologous. The promoters can be either native or synthetic. Native promoters and genes can be amplified from yeast genomic DNA. Vectors with bi-directional double promoter cassettes can be obtained from AddGene.<br>The cloning process consists of: a) vector preparation, b) primer design, c) generation of gene and promoter biobricks via PCR, d) USER cloning. The vectors should be confirmed by sequencing. The procedures are described in details on the p. 7-11.
**Transformation into yeast**	Using integrative vectors without a selection marker, and non-integrative gRNA helper vectors with nourseothricin selection marker, and a Cas9 expression vector with G418 selection marker, one to three vectors can be transformed into yeast simultaneously.<br>The transformation procedure is described on p. 13.
**Removal of the selection markers**	After a successful transformation, it is necessary to lose the gRNA helper vector. This is achieved by growing the selected transformant overnight on a YPD agar plate containing only G418 to keep the Cas9 expression vector. Routinely, ~90% of the colonies will have lost the gRNA helper vector. This can be confirmed by replica plating on YPD with G418 and nourseothricin.

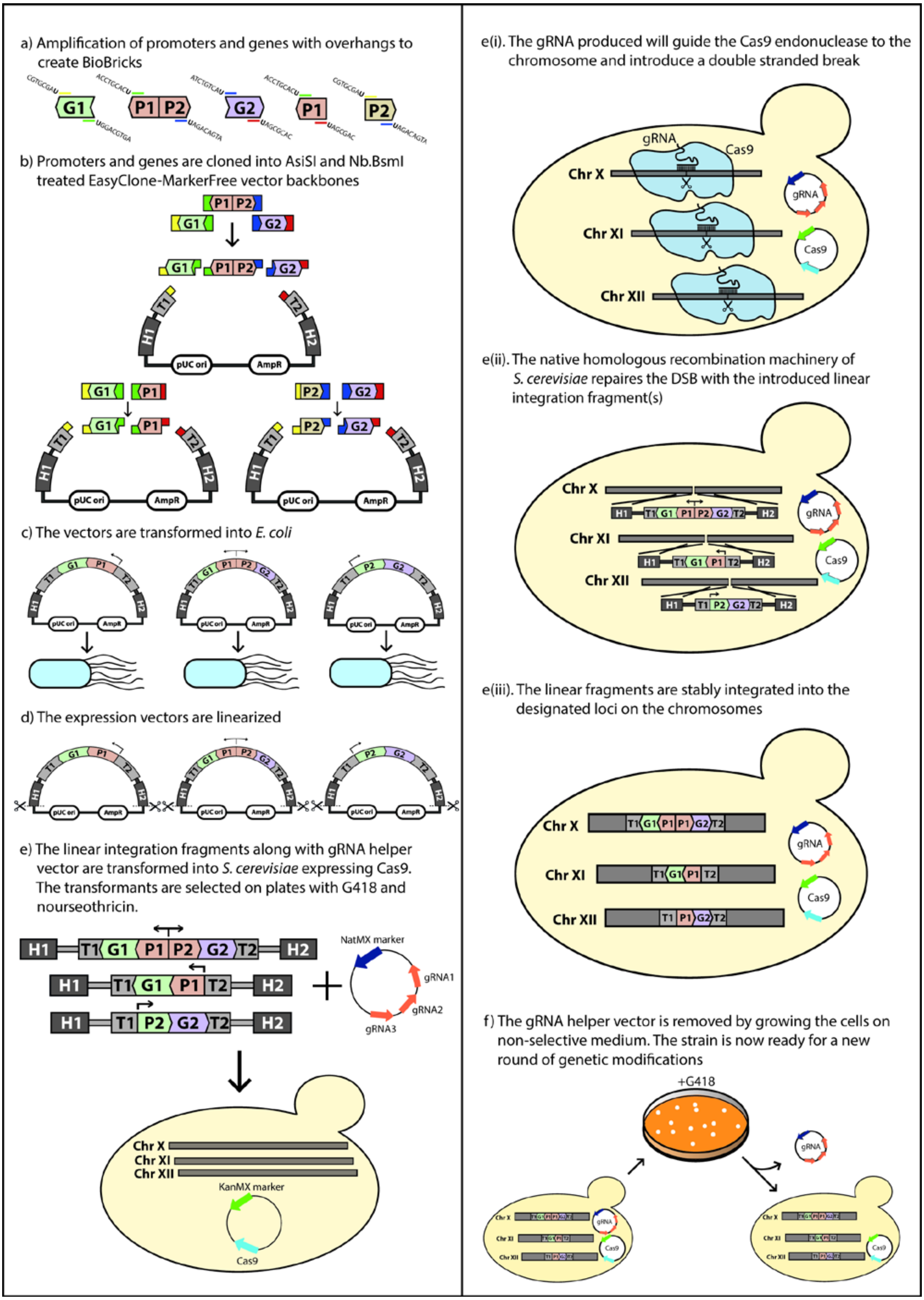
a) Amplification of promoters and genes with overhangs to create BioBricks
G1
P1
P2
G2
b) Promoters and genes are cloned into AsiSI and Nb.BsmI treated EasyClone-MarkerFree vector backbones
T1
T2
H1
H2
pUC ori
AmpR
c) The vectors are transformed into E. coli
d) The expression vectors are linearized
e) The linear integration fragments along with gRNA helper vector are transformed into S. cerevisiae expressing Cas9. The transformants are selected on plates with G418 and nourseothricin.
NatMX marker
gRNA1
gRNA2
gRNA3
Chr X
Chr XI
Chr XII
KanMX marker
Cas9
e(i). The gRNA produced will guide the Cas9 endonuclease to the chromosome and introduce a double stranded break
gRNA
e(ii). The native homologous recombination machinery of S. cerevisiae repaires the DSB with the introduced linear integration fragment(s)
e(iii). The linear fragments are stably integrated into the designated loci on the chromosomes
f) The gRNA helper vector is removed by growing the cells on non-selective medium. The strain is now ready for a new round of genetic modifications
+G418

PROTOCOLS

Preparation of vectors for USER cloning

**Digestion with AsiSI/SfaAI**	The EasyClone-MarkerFree integrative vectors are linearized with SfaAI (AsiSI), e.g. FastDigest® SfaAI from ThermoScientific.<br><br>1. Prepare the reactions as following:<br>X µl of EasyClone 2.0 vector (20 µg)<br>20 µl of FastDigest® buffer<br>5 µl of FastDigest SfaAI® restriction enzyme<br>Water to a final volume of 200 µl<br>2. Incubate for 1 hour at 37°C.<br>3. Purify the plasmid from solution, using e.g., NucleoSpin® Gel and PCR Clean-up from Macherey Nagel, eluting with 50 µl of elution solution.<br>4. Determine DNA concentration using Nanodrop or sim.<br><br>Proceed to nicking step.
**Nicking with Nb.BsmI**	The vectors linearized with SfaAI (AsiSI) are nicked with Nb.BsmI, e.g. from New England Biolabs Inc.<br><br>1. Prepare the reactions as following:<br>40 µl of SfaAI-digested vector<br>X µl of Nb.BsmI (use 1U per 1 µg of vector)<br>5 µl of buffer NEB 3.1<br>2. Incubate for 1 hour at 65°C (best use PCR machine and heated lid protocol, otherwise the water will evaporate from the reaction mix and condense on the lid).<br>5. Purify the digested and nicked vector from the gel, using e.g., NucleoSpin® Gel and PCR Clean-up from Macherey Nagel. Elute with 50 µl of elution solution.<br>3. Determine DNA concentration using Nanodrop or sim.<br>4. Store the USER-ready vectors at -20°C for repeated use.<br><br>Proceed to USER cloning.

Primer design

Primers with uracil overhangs

Design primers for amplification of your genes and promoters of interest with the appropriate overhangs (p. 6, a).
The primers must contain uracils and can be ordered from commercial oligo suppliers, e.g., IDT DNA.

The primers should contain the following overhangs:

Gene 1 primers
forward primer (GP1F): **AGTGCAGGU** *AAAACA* ATG(N)$_n$
reverse primer (GV1R): **CGTGCGAU** TCA(N)$_n$

Gene 2 primers
forward primer (GP2F): **ATCTGTCAU** *AAAACA* ATG(N)$_n$
reverse primer (GV2R): **CACGCGAU** TCA(N)$_n$

Promoter 1 primers
forward primer (PV1F): **CACGCGAU**(N)$_n$
reverse primer (PG1R): **ACCTGCACU(**N)$_n$

Promoter 2 primers
forward primer (PV2F): **CGTGCGAU**(N)$_n$
reverse primer (PG2R): **ATGACAGAU**(N)$_n$

USER overhang in bold, *AAAACA* – Kozak sequence, ATG – start codon, TCA – stop codon, (N)$_n$ – gene(promoter)-specific sequence.

Preparation of DNA BioBricks

**Uracil-tolerant polymerases**	BioBricks, encoding genes and promoters, are amplified with primers containing uracils (p. 6, a). It is necessary to use a DNA polymerase that can read through uracils present in the primers and that has proofreading activity, e.g., Phusion U Hot Start DNA Polymerase from ThermoFisher Scientific or PfuTurbo Cx Hotstart DNA Polymerase from Agilent. Follow the manufacturer's instructions for the PCR conditions.
**PCR-amplification of BioBricks**	1. Prepare the PCR reactions as following:<br>32 µl of water<br>10 µl of 5X Phusion HF Buffer<br>2.5 µl of forward primer (10 µM)<br>2.5 µl of reverse primer (10 µM)<br>1 µl of dNTP mix (10 mM)<br>1 µl template DNA (plasmid or genomic DNA)<br>1 µl of Phusion U Hot Start DNA Polymerase<br>2. Run the following PCR program:<br>98°C for 1 min<br>30 cycles of<br>[98°C for 10 seconds<br>54°C for 30 seconds (or another suitable annealing temperature)<br>72°C for 1 min per 1 kb of the PCR product]<br>72°C for 5 min<br>10°C pause<br>3. Purify the BioBricks from the gel using e.g., NucleoSpin® Gel and PCR Clean-up from Macherey Nagel, eluting with 50 µl of elution solution. *Note: alternatively the BioBricks can be purified from solution or PCR reaction can be used directly. This can however result in lower cloning efficiency.*<br>4. Store the BioBricks at -20°C for repeated use.<br><br>Proceed to USER cloning.

USER cloning of BioBricks into vector

USER cloning and transformation into *E. coli*

The Biobricks are cloned into EasyClone 2.0 vectors, treated with SfaAI/Nb.BsmI (p. 6, b).

1. Prepare the USER reaction as following:
   1 µl of SfaAI/Nb.BsmI-treated vector*
   1 µl of BioBrick for gene 1   use vector to insert molar ratio 1:3
   1 µl of BioBrick for gene2
   1 µl of BioBrick for double promoter
   0.5 µl 5xHF buffer (NEB)
   0.5 µl USER™ enzyme (NEB)

   Note: when cloning only gene1 or gene2, use water instead of the missing BioBrick.

   *(*Optional step – you can test the quality of the vector digestion before you proceed to the cloning of a gene of interest*
   - Clone a gene encoding green fluorescent protein suitable for expression in E. coli into a SfaAI/Nb.BsmI-treated vector
   - The GFP gene can be amplified from p1976 (pUC19-GFP-cassette) vector with the following primers: CGTGCGAUCCGCATAGGGAGTGTAAATTTATC
   CACGCGAUAGTGAAAGGAAGGCCCATGAG
   - After you have finished the steps 2 and 3, evaluate the number of background (white) transformants on plates under blue light bench top illuminator – at least 80% of positive (green) colonies should be obtained)
2. Incubate the mixture in PCR machine at the following conditions:
   37°C for 25 min
   25°C for 10 min
   20°C for 10 min
   15°C for 10 min
   10°C pause
3. Transform the reaction mix into competent *E. coli* cells.
   - Cool the tubes on ice and add 95 µl of competent *E. coli* DH5α cells
   - After 10 min on ice, perform heat shock at 42°C for 90 s and place the tubes on ice for 1-2 min
   - Add 50 µl SOC media to each tube and incubate at 37°C incubator for 30 min
   - Plate the cells on LB-amp plates and incubate at 37°C overnight.

Proceed to verification of expression vector assembly.

Verification of expression vector assembly by colony PCR

Verification primers

The successful cloning of genes and promoters into the EasyClone-MarkerFree integrative vectors can be identified by PCR on *E. coli* colonies.
If the total size of the cloned fragments does not exceed 5 kb, then the following primers can be used:

- ADH1_test_fw: GAAATTCGCTTATTTAGAAGTGTC
- CYC1_test_rv: CTCCTTCCTTTTCGGTTAGAG

In the opposite case a pair of primers can be used to verify cloning of gene1: forward primer used for amplification of the gene1 and ADH1_test_fw as a reverse primer
and cloning of gene2: forward primer used for amplification of the gene2 and CYC1_test_rv as a reverse primer

***E. coli* colony PCR**

1. Mix the following in a PCR tube (for 10 reactions of 10 µl each):
   50 µl 2xOneTaq Master Mix (New England Biolabs)
   10 µl µM forward verification primer
   10 µl µM reverse verification primer
   30 µl water
2. Add small amount of *E. coli* colony biomass (it is enough to touch the colony with a tip) to each PCR tube.
3. Run the following PCR program:
   94°C for 3 min
   35 cycles of
   [94°C for 20 seconds
   50°C for 30 seconds (or another suitable annealing temperature)
   68°C for 1 min per 1 kb of the PCR product]
   68°C for 5 min
   10°C pause
4. Analyze the PCR reactions on 1% agarose gel or on LabChip GXII (Caliper).

Plasmid purification and sequencing

1. Inoculate the *E. coli* clones containing correct vectors into 3-5 ml of liquid LB medium with ampicillin and cultivate overnight at 37°C.
2. Preserve the *E. coli* for future use: mix 500 µl of overnight *E. coli* culture with 500 µl of 50% v/v sterile glycerol solution. Store in cryotubes at -80°C.
3. Use the rest of the overnight culture to purify the vectors (e.g. NucleoSpin kit from Macherey-Nagel).
4. Sequence the cloned inserts.

Proceed to vector linearization and transformation.

Linearization of expression vector

**Choice of restriction enzyme for linearization**	Before transformation into yeast, the constructed expression vectors are linearized. Linearization of the vectors promotes integration into chromosome via homologous recombination. The vector fragments are integrated via double cross-over events of yeast chromosome with the UP and DOWN regions, flanking the expression cassette.<br><br>The DNA fragment targeted for integration can be excised from the expression vector by digestion with NotI.<br>If however any of the cloned genes or promoters contain NotI recognition sequence (GC^GGCC_GC), then other restriction enzymes should be used, e.g., SmiI (SwaI).
**Linearization with NotI**	1. Mix the following:<br>X µl of expression vector (min 1 µg)<br>5 µl of FastDigest® buffer<br>X µl of FastDigest NotI® (use 0.2 µl per 1 µg DNA)<br>water up to 50 µl<br>2. Incubate at 37°C for 1 hour.<br>3. (Optional step) Confirm linearization on the gel and if desired purify the correct fragment from the gel (be aware of the *E. coli* backbone fragment of 2.8 kb).<br>4. Purify the linearized vector from solution using e.g., NucleoSpin® Gel and PCR Clean-up from Macherey Nagel. Store at -20°C until transformation.

Transformation of expression vector(s) into yeast (according to Gietz and Woods, 2002)

Preparation of competent *S. cerevisiae* cells

1. Inoculate the yeast strain into 10 ml of YPD or SC selection medium with 200 mg/L G418 and grow overnight at 30°C in shaker.
   Note: For integrative transformations, the strain selected must have been previously transformed with the Cas9 expression vector using the following protocol
2. Determine the titer of the yeast culture and inoculate 2.5 x 10^8 cells into 50 ml of fresh YPD medium to give 5x10^6 cells per ml of the culture.
3. Grow the culture for approx. 4 hours to get the cell titer 2x10^7 cells per ml
4. Harvest the cells by centrifugation, wash in 25 ml of sterile water and resuspend in 1 ml water.
5. Transfer the cell suspension to a 1.5 ml microcentrifuge tube, spin down for 30 sec and discard the supernatant.
6. Add water to a final volume of 1.0 ml and vortex mix vigorously to resuspend the cells.
   Note: If the cell count of the culture is greater than 2 x 10^7 cells per ml the volume then increase the volume to maintain the titer of this suspension at 2 x 10^9 cells per ml. If the cell count of the culture is less than 2 x 10^7 cells/ml then decrease the volume.
7. Pipette 100 µl samples (10^8 cells) into 1.5 ml microfuge tubes, one for each transformation, centrifuge at top speed for 1 min and remove the supernatant.

Proceed to yeast transformation.

Yeast transformation and selection

1. Mix the following Transformation mix sufficient:
   240 µl 50% (w/v) Polyethylene glycol MW 3350 (PEG)
   36 µl 1M Lithium Acetate (LiAc)
   10 µl ss-carrier DNA (10 mg/ml) – boil for 3 min before use and keep on ice
   0.1-1 µg linearized integration vector (or Cas9 expression vector)
   0.5 µg gRNA helper vector (do not add if transforming with Cas9 vector)
   Add water to a final volume of 360 µl
2. Keep the Transformation Mix in ice water until mixed with competent cells.
3. Add 360 µl of Transformation Mix to each transformation tube and resuspend the cells by vortex mixing vigorously.
4. Incubate the tubes in a 42°C water bath for 40 min.
5. Spin down the cells, remove the supernatant and resuspend the cells in 1 ml YPD without antibiotics.
6. Incubate the cells in YPD for 2 hours at 30°C with shaking to provide time for expression of a resistance gene.
7. Spin down the cells, resuspend in water and plate on YPD agar plates containing 200 mg/L G418 and 100 mg/L nourseothricin.

Proceed to verification of correct vector integration in the yeast genome.

**Removal of the gRNA helper vector**	1. Select a confirmed correct transformant<br>2. Plate the verified colony onto a YPD agar plate containing 200 mg/L G418 and incubate overnight at 30°C<br>3. The following day replica plate onto a YPD agar plate containing 200 mg/L G418 and 100 mg/L nourseothricin and incubate overnight at 30°C<br>4. Colonies that have not grown on the replica plate are confirmed to have successfully lost the gRNA helper vector and can be used for subsequent modifications

APPENDIX

Verification of correct vector integration in the genome by yeast colony PCR

Verification primers and expected fragment sizes

Select suitable primers from the table below for verification of a correct insertion of your construct.

Note: Primers 2220 and 2221 are universal primers annealing to any vector of choice. The other primers are from a particular genomic region. Either one or both primer pairs can be used for verification of the particular correct integration.

Site	Primer ID	Sequence of the primer 5´-3´	Description	Fragment size (bp)
X-2	2220	CCTGCAGGACTAGTGCTGAG	X-2 DOWN	973
	902	GAGAACGAGAGGACCCAACAT		
	2221	GTTGACACTTCTAAATAAGCGAATTTC	X-2 UP	873
	901	TGCGACAGAAGAAAGGGAAG		
X-3	2220	CCTGCAGGACTAGTGCTGAG	X-3 DOWN	667
	904	CCGTGCAATACCAAAATCG		
	2221	GTTGACACTTCTAAATAAGCGAATTTC	X-3 UP	1059
	903	TGACGAATCGTTAGGCACAG		
X-4	2220	CCTGCAGGACTAGTGCTGAG	X-4 DOWN	656
	906	GACGGTACGTTGACCAGAG		
	2221	GTTGACACTTCTAAATAAGCGAATTTC	X-4 UP	983
	905	CTCACAAAGGGACGAATCCT		
XI-1	2220	CCTGCAGGACTAGTGCTGAG	XI-1 DOWN	784
	908	GAAGACCCATGGTTCCAAGGA		
	2221	GTTGACACTTCTAAATAAGCGAATTTC	XI-1 UP	791
	907	CTTAATGGGTAGTGCTTGACACG		
XI-2	2220	CCTGCAGGACTAGTGCTGAG	XI-2 DOWN	818
	910	GAGACAAGATGGGGCAAGAC		
	2221	GTTGACACTTCTAAATAAGCGAATTTC	XI-2 UP	963
	909	GTTTGTAGTTGGCGGTGGAG		
XI-3	2220	CCTGCAGGACTAGTGCTGAG	XI-3 DOWN	704
	912	CACATTGAGCGAATGAAACG		
	2221	GTTGACACTTCTAAATAAGCGAATTTC	XI-3 UP	927
	911	GTGCTTGATTTGCGTCATTC		
XI-5	2220	CCTGCAGGACTAGTGCTGAG	XI-5 DOWN	701
	8419	GCATGGTCACCGCTATCAGC		
	2221	GTTGACACTTCTAAATAAGCGAATTTC	XI-5 UP	1050
	8418	CTCAATGATCAAAATCCTGAATGCA		
XII-1	2220	CCTGCAGGACTAGTGCTGAG	XII-1 DOWN	896
	892	GGACGACAACTACGGAGGAT		
	2221	GTTGACACTTCTAAATAAGCGAATTTC	XII-1 UP	852

	891	CTGGCAAGAGAACCACCAAT		
XII-2	2220	CCTGCAGGACTAGTGCTGAG	XII-2 DOWN	666
	894	GGCCCTGATAAGGTTGTTG		
	2221	GTTGACACTTCTAAATAAGCGAATTTC	XII-2 UP	795
	893	CGAAGAAGGCCTGCAATTC		
XII-3	2220	CCTGCAGGACTAGTGCTGAG	XII-3 DOWN	744
	896	TGGCCAATTGTTCAGTCAAG		
	2221	GTTGACACTTCTAAATAAGCGAATTTC	XII-3 UP	963
	895	TGGGCAGCCTTGAGTAAATC		
XII-4	2220	CCTGCAGGACTAGTGCTGAG	XII-4 DOWN	667
	898	CGTGAAATCTCTTTGCGGTAG		
	2221	GTTGACACTTCTAAATAAGCGAATTTC	XII-4 UP	828
	897	GAACTGACGTCGAAGGCTCT		
XII-5	2220	CCTGCAGGACTAGTGCTGAG	XII-5 DOWN	799
	900	GTGGGAGTAAGGGATCCTGT		
	2221	GTTGACACTTCTAAATAAGCGAATTTC	XII-5 UP	811
	899	CCACCGAAGTTGATTTGCTT		

Yeast colony PCR

1. Check the transformation plates after 2-5 days of growth at 30°C.
2. Pick several clones and streak on selective plate.
3. Take small amount of fresh biomass and resuspend in 15 µl of 20 mM NaOH.
4. Incubate for 15 min at 96°C.
5. Vortex briefly and spin down the cells.
6. Mix the following in a PCR tube (for 10 reactions):
   50 µl 2xOneTaq Master Mix (New England Biolabs)
   10 µl µM primer 1
   10 µl µM primer 2
   20 µl water
7. Add 1 µl of the denatured biomass to each tube containing 9 µl of the PCR premix.
8. Run the following PCR program:
   94°C for 1 min
   35 cycles of
   [94°C for 20 seconds
   50°C for 30 seconds
   68°C for 1 min/kb of the PCR product]
   68°C for 7 min
   10°C pause
9. Analyze the samples on 1% agarose gel or on LabChip GXII (Caliper) (for corresponding PCR product size see the Table above).

Media recipes

**YPD (+antibiotics)**	Yeast Extract Peptone Dextrose Medium (1 liter)<br>1% yeast extract<br>2% peptone<br>2% dextrose (glucose or galactose)<br>± antibiotics for selection in the following concentrations:<br>200 mg/l G418 (kanMX selection marker)<br>100 mg/l Nourseothricin (natMX selection marker)<br>± 2% agar<br><br>1. Dissolve the following in 900 ml of water:<br>10 g yeast extract<br>20 g of peptone<br>(for plates) add 20 g of agar<br>2. Autoclave for 20 min at 120°C.<br>3. Cool solution to ~60°C and add 100 ml of 20% glucose.<br>4. For YPD agar, add antibiotics, if desired, and pour the plates.<br><br>Liquid YPD can be stored at room temperature. Antibiotics are added immediately prior to use.<br>YPD plates are stored at 4°C in the dark for up to one month.
**SC**	Synthetic Complete Medium<br>Yeast Nitrogen Base without aminoacids (Sigma-Aldrich catalogue number Y0626)<br>Yeast Synthetic Drop-out Medium Supplements<br>w/o uracil (Y1501)<br>w/o histidine (Y1751)<br>w/o lysine (Y1896)<br>w/o leucine (Y1376)<br>2% dextrose (glucose)<br>± antibiotics for selection in the following concentrations:<br>200 mg/l G418 (kanMX selection marker)<br>100 mg/l Nourseothricin (natMX selection marker)<br>± 2% agar<br><br>For exact composition and protocol please visit the products' website<br><br>Liquid SC medium can be stored at room temperature. Plates should be stored at 4°C.
**LB-amp**	Lysogeny Broth Medium (1 liter)<br>1% tryptone<br>0.5% yeast extract<br>1% NaCl<br>1.5% agar for LB agar<br>1. Dissolve the following in 1 liter of water:<br>10 g tryptone<br>5 g yeast extract

10 g NaCl
for LB agar add 15 g agar

2. Autoclave for 20 min at 120°C.
3. Cool solution to ~60°C and add 100 mg/l ampicillin.
4. Poor the plates in case of LB agar medium.

The liquid as well as solid medium containing ampicillin should be stored at 4°C.

SOC

Super Optimal Broth (250 ml)
0.5 % yeast extract
2 % tryptone
10 mM NaCl
2.5 mM KCl
10 mM $MgCl_2$
10 mM $MgSO_4$
20 mM glucose

To make 250 mL of media:

1. Dissolve the following in 231 ml of water:
   1.25 g yeast extract
   5 g tryptone
   0.15 g NaCl
   0.005 g KCl
   0.51 g $MgCl_2$
2. Adjust pH to 7 by adding sodium hydroxide.
3. Autoclave the solution at 120°C for 20 min.
4. Let the solution cool and then add 10 ml of 20% glucose and 2.5 ml of 1M $MgSO_4$

Medium can be stored at room temperature.

REFERENCES

Gietz RD and Woods RA (2002) Transformation of yeast by the LiAc/SS carrier DNA/PEG method. Methods in Enzymology 350: 87-96

Hegemann JH and Heick SB (2011) Delete and repeat: a comprehensive toolkit for sequential gene knockout in the budding yeast *Saccharomyces cerevisiae*. Methods Mol Biol 765: 189-206

Jensen NB, Strucko T, Kildegaard KR, David F, Maury J, Mortensen UH, Forster J, Nielsen J, Borodina I (2014) EasyClone: method for iterative chromosomal integration of multiple genes in Saccharomyces cerevisiae. FEMS Yeast Research 14:238–248

Jessop-Fabre MM, Jakočiūnas T, Stovicek V, Dai Z, Jensen MK, Keasling J, Borodina I (2015) EasyClone-MarkerFree: A vector toolkit for marker-less integration of genes into *Saccharomyces cerevisiae.* (Submitted)

Mikkelsen MD, Buron LD, Salomonsen B, Olsen CE, Hansen BG, Mortensen UH, Halkier BA (2012) Microbial production of indolylglucosinolate through engineering of a multi-gene pathway in a versatile yeast expression platform. Metabolic Engineering 14:104–111

Stovicek V., Borja G., Forster J., Borodina I. (2015): EasyClone 2.0: Expanded toolkit of integrative vectors for stable gene expression in industrial Saccharomyces cerevisiae strains. Journal of Industrial Microbiology and Biotechnology
